# Biomimetic metamaterial–based interface for decoding heterogeneous mechanodermal activity

**DOI:** 10.1126/sciadv.aee2625

**Published:** 2026-04-22

**Authors:** Muzi Xu, Jiaqi Zhang, Chaoqun Dong, Zibo Zhang, Duanyang Li, Wentian Yi, Miaomiao Zou, Chenyu Tang, George G. Malliaras, Luigi G. Occhipinti

**Affiliations:** ^1^ Electrical Engineering Division, Department of Engineering, University of Cambridge, Cambridge CB3 0FA, UK.; ^2^ Department of Electrical and Electronic Engineering, University of Hong Kong, Pokfulam Road, Hong Kong SAR 999077, China.; ^3^ Institute for Manufacturing, Department of Engineering, University of Cambridge, Cambridge CB3 0FS, UK.

## Abstract

The human skin acts as a dynamic biomechanical interface that conveys critical physiological and behavioral information through spatiotemporally distributed deformations. Because of the limited capabilities of current sensing technologies, the spatiotemporal diversity of its mechanical cues has remained underused to date, preventing these mechanisms from being used to capture and decode the full spectrum of underlying physiological states. In this work, we define this heterogeneous set of mechanical signals as mechanodermal activity (MDA) and introduce the biomimetic metamaterial–based interface (BMMI), an engineered auxetic metamaterial substrate that reproduces the microrelief and mechanoreceptor architecture of natural skin. The BMMI allows selective capture of diverse MDA signals from adjacent skin regions with simultaneous signal amplification and noise suppression and permits straightforward modulation to accommodate various scenarios. Combined with bespoke algorithms, the wireless BMMI device decodes MDA accurately and robustly for multimodal communication interfaces, unleashing applications in health care monitoring and human-machine interaction.

## Teaser

A biomimetic metamaterial–based interface with selective sensing decodes heterogeneous mechanodermal activity.

## INTRODUCTION

The human skin serves as a dynamic biomechanical interface, reflecting a wide spectrum of mechanical deformations corresponding to physiological changes within the body. These deformations, spatiotemporally distributed across the skin, encode a rich array of biological information that provides noninvasive access to key indicators of physiological states and behaviors (*1*–*7*). Recent research has predominantly focused on capturing and analyzing single classes of biomechanical deformations, such as tracking hand movements for rapid gesture recognition (*6*, *8*) or detecting subtle arterial pressure for cardiac function assessment (*9*, *10*). However, these approaches overlook the heterogeneous and spatially distributed nature of mechanical signals across adjacent skin regions, limiting the possibility of decoding the full spectrum of physiological information that can be obtained from skin activity, hindering a comprehensive understanding and automatic decoding of the body’s dynamics and body language. To address this fundamental aspect, we define the property of the human body that causes the spatiotemporal distribution of mechanical deformations across the skin as mechanodermal activity (MDA), a unifying framework that integrates heterogeneous mechanical signals and opens promising avenues in health care monitoring, disease diagnosis, and human-machine interaction.

While electrodermal activity has been widely studied and correlated to electrical activity occurring in the bulk, as in the case of electromyography used to decode muscle activity, MDA occurs dynamically on the skin surface, where deformation of adjacent regions of skin produces signals and information with distinct characteristics. Taking the neck region as an example, spatially distributed heterogeneous signals, including small vibrations from the carotid artery, large deformations from lateral muscle movement, and small vibrations from laryngeal activity, can be captured simultaneously, providing physiological information associated with nonverbal communication (*11*). Existing strain sensors on flexible and conformable substrates have primarily emphasized improvements in overall sensitivity, extending their single-point signal detection sensitivity by using advanced nanomaterials (*12*–*14*) or innovative structures (*15*–*18*). These strategies, however, fail to capture the human skin’s natural ability to produce spatially selective responses and parallel multisignal processing (*19*) to directly capture high-fidelity signals at the source while simultaneously amplifying small vibrations and suppressing noise from large deformations across the skin. To effectively decode these heterogeneous MDAs, next-generation interfaces need to feature spatially selective signal acquisition capabilities with high specificity for both small vibrations and large deformations.

## RESULTS

### Biomimetic metamaterial–based interface (BMMI)

**Fig. 1.**
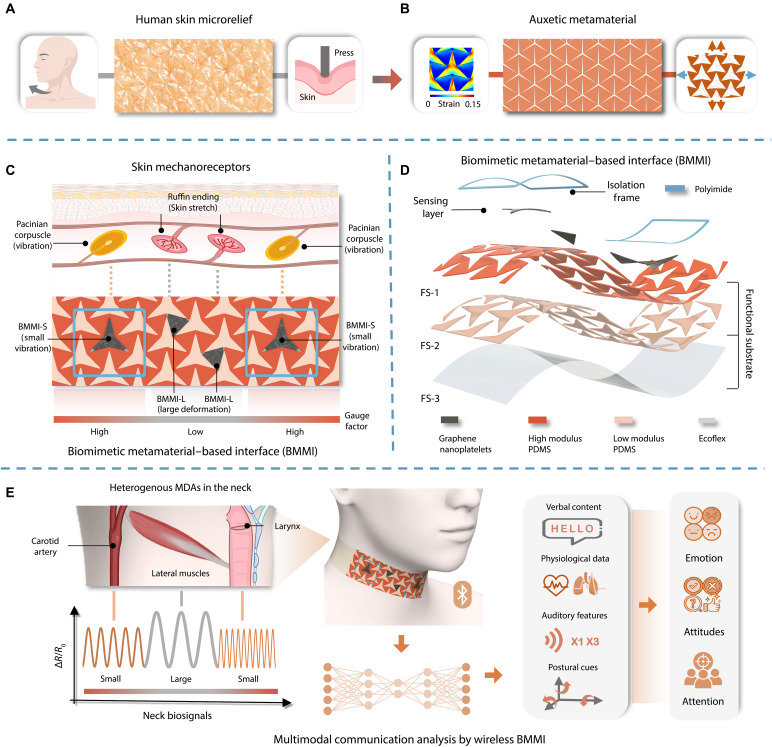
Overview of the BMMI design. (**A**) Photograph of the human skin microrelief, which accommodates large strains induced by body movements and generates localized strain concentrations under compression. (**B**) Auxetic metamaterial featuring star-shaped perforations, facilitating strain redistribution upon stretching. (**C**) The BMMI mimics the targeted tactile capabilities of human skin. BMMI-S and BMMI-L correspond to skin mechanoreceptors, namely Pacinian corpuscles and Ruffini endings, enabling the selective detection of small vibrations and large deformations, respectively. (**D**) The structure of the BMMI consists of a functional substrate (FS-1, FS-2, and FS-3), a sensing layer, and isolation frames. (**E**) Multimodal communication analysis using a bespoke deep learning–based model with four-channel heterogeneous data collected by a wireless BMMI attached to the neck region, enabling accurate recognition of cognitive and affective states. Some elements in schematics in (C) and (E) were created in BioRender. Shepherd, K. (2026), https://biorender.com/7yep69g, https://biorender.com/qe7wjfx.

Here, we introduce a BMMI designed to achieve amplification of small vibrations and noise suppression of large deformations at the hardware level. This dual functionality is obtained through a biomimetic design approach incorporating an auxetic metamaterial, with dual tunable gauge factor (GF) and detection limit, producing heterogeneous responses to multiple MDAs in the same substrate (Fig. 1).

The BMMI mimics human skin in both surface morphology and tactile functionality. Skin microrelief refers to the intricate microscopic topography of the skin surface (*20*), composed of a network of furrows and ridges that form plateaux with triangular shapes (*21*). As illustrated in Fig. 1A, this microrelief enhances the skin’s stretchability, allowing it to accommodate large strains induced by body movements without mechanical failure or tearing. Furthermore, the microrelief induces localized strain concentrations under compression, amplifying sensitivity to mechanical stimuli while attenuating responses in adjacent regions (*21*, *22*). Inspired by this natural design, we implemented an auxetic metamaterial featuring star-shaped perforations as the structural backbone and applied strain engineering to create a heterogeneous functional substrate by modulating Young’s modulus (Fig. 1B). Upon stretching, this architecture facilitates strain redistribution across the substrate. The star-shaped perforations filled with low-modulus materials promote localized strain concentration, while the high-modulus triangular backbone enables strain relaxation. This auxetic design also ensures excellent conformability to the skin’s nonzero Gaussian surface.

Building on its biomimetic surface morphology, the BMMI substrate integrates regions of both strain concentration and relaxation. As shown in Fig. 1C, the BMMI leverages the distinct properties of these two regions to replicate the tactile capabilities of human skin, achieving targeted sensing of both small vibrations, through BMMI-S, and large deformations, through BMMI-L, which correspond to the functions of Pacinian corpuscles and Ruffini endings, respectively. This design is directly inspired from the multimodal sensory system of human skin, which comprises low-threshold mechanoreceptors with unique sensitivities and adaptive properties, enabling humans to perceive and respond to mechanical stimuli from the physical environment with high specificity (*19*, *23*). Pacinian corpuscles, acting as fast-adapting receptors, are responsible for measuring vibrations transmitted through tissues, whereas Ruffini endings, as slowly adapting receptors, primarily respond to skin stretching as a result of tensile strain applied to the skin (*24*, *25*). Accordingly, BMMI-S, located in the low-modulus regions, exhibits a high GF, allowing precise detection of subtle skin vibrations. In contrast, BMMI-L, situated in the high-modulus regions, has a low GF, ensuring accurate sensing of large deformations. By seamlessly integrating heterogeneous mechanical responses within a single substrate, this biomimetic design creates a simple, efficient, and tunable interface that selectively amplifies small vibrational signals while inherently suppressing excessive mechanical noise from large deformations, hence offering a unique and promising strategy for versatile and efficient decoding of diverse MDAs.

In Fig. 1D, the exploded view depicts the structure and material composition of the fabricated BMMI, which consists of three main components: isolation frames, a sensing layer, and a functional substrate (FS-1, FS-2, and FS-3). FS-1, serving as the structural backbone, is designed as an auxetic metamaterial with star-shaped perforations and exhibits a high modulus optimized for responding to large deformations. In contrast, FS-2, designed for detecting small vibrations, is formed by filling the star-shaped perforations of FS-1 with a low-modulus material, which results in a continuous, integrated functional layer. FS-3, with a tissue-like modulus, serves the purpose of a bottom layer optimized for direct conformal contact with the skin to facilitate strain transduction. Last, the graphene nanoplatelet–based sensing layer is deposited onto the surfaces of FS-1 and FS-2, forming the BMMI-L and BMMI-S sensors, respectively. A detailed fabrication procedure and cross-sectional schematic of the complete BMMI architecture are provided in figs. S1 and S2.

Using the fabricated BMMI integrated with a wireless electronics module, we demonstrated its effectiveness in multimodal communication analysis applications (Fig. 1E). In the area of wearable electronics, conventional communication interfaces primarily focus on recognizing verbal information collected from the throat or mouth regions (*2*, *26*, *27*). While these systems perform well in word identification and sentence decoding, they often neglect the nonverbal cues that are essential for a natural and effective human interaction. To overcome the limitations of these single-modality systems and enable a more comprehensive understanding of communicative intent, the BMMI was applied to the neck region to selectively capture spatiotemporally distributed, heterogeneous MDAs occurring in this region. In addition to verbal content, the BMMI simultaneously captured a wide array of nonverbal signals, including physiological data (pulse and respiration), auditory features (volume and speech rate), and postural cues linked to muscle activity. These signals, typically acquired using multiple sensors placed at various body sites, were now collected by a single device. The four-channel BMMI system wirelessly transmitted these heterogeneous signals, which were further decoded using a bespoke deep learning model to extract underlying cognitive and affective states such as attention, attitude, and emotion. As a result, the system achieved high-fidelity multimodal signal acquisition and enabled accurate recognition of these states with up to 95% accuracy, supporting more profound understanding and more effective communication.

### Modulation mechanism of the metamaterial-based substrate

Mechanical metamaterials are artificially designed materials with engineered architectures that demonstrate properties surpassing those of conventional materials (*28*–*30*). To break the existing fetters, these high-performance metamaterials have been widely applied in the field of flexible electronics for enhancing and innovating functionalities (*31*–*33*). Among them, auxetic metamaterials, exhibiting a negative Poisson’s ratio, offer superior biaxial stretchability, improved conformability to curved surfaces, and greater tunability compared to traditional elastomers (*33*–*35*). Building on these advantages, an auxetic metamaterial with star-shaped perforations is used in this work to modulate the strain distribution in BMMI. Unlike other auxetic structures, such as reentrant or chiral designs, the rotating triangle geometry provides sufficient space for both the high-modulus metamaterial backbone and perforations filled with low-modulus material to accommodate the sensing materials. As a result, such a biomimetic metamaterial–based substrate, integrated with strain engineering (*36*), achieves strain concentration in low-modulus regions and strain relaxation in high-modulus areas, enabling the selective detection of heterogeneous biophysical signals.

**Fig. 2.**
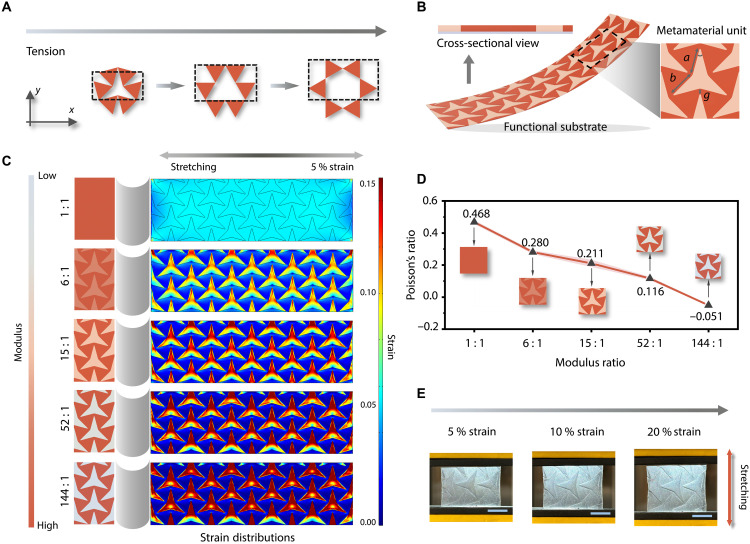
Modulation mechanism of the metamaterial-based substrate. (**A**) Mechanism of auxetic behavior with rotating rigid triangles. Under tensile strain, these triangles rotate, forming a more open structure and achieving a negative Poisson’s ratio. (**B**) Cross-sectional view and metamaterial unit of the BMMI’s functional substrate. (**C**) FEA results of functional substrates with five different modulus ratios between FS-1 and FS-2 (1:1, 6:1, 15:1, 52:1, and 144:1) under 5% strain. (**D**) Poisson’s ratios of the five functional substrates, fabricated with varying modulus ratios. (**E**) Photographs of the BMMI’s functional substrate under different tensile tests. Scale bars, 1 cm.

Figure 2A describes the mechanism of auxetic behavior with rotating rigid triangles. Under tensile strain, these triangles begin to rotate, resulting in an increased angle between them, which forms a more open structure and achieves a negative Poisson’s ratio. Leveraging this mechanism, we innovatively replaced the rigid triangles with high-modulus stretchable polydimethylsiloxane (PDMS) and filled the star-shaped perforations with low-modulus PDMS. This dual-modulus design not only retains the auxetic metamaterial’s capability to modulate the Poisson’s ratio but also converts the entire functional substrate into a flexible, stretchable interface exhibiting heterogeneous mechanical responses. As shown in Fig. 2B, the cross-sectional view demonstrates that the high-modulus FS-1 and low-modulus FS-2 together form a continuous, integrated functional layer comprising multiple metamaterial units with tunable geometric parameters. Specifically, as the *r* parameter, defined as the ratio of side lengths *a* to *b*, approaches the value of 1, the auxetic behavior of the metamaterial is enhanced (*37*). To maximize the auxetic performance of the functional substrate, *r* is therefore set to 1, corresponding to an equilateral triangular geometry. Previous studies have demonstrated that noncrystalline systems can exhibit auxetic behavior comparable to that of rigid triangle-based designs, which is best achieved at lower values of the hinging angle (θ) and the spacing (*g*) between adjacent star-shaped regions (*37*). On the basis of this insight, the dimensions of our metamaterial units were defined as shown in fig. S3, where θ is 30° and *g* is 1.5 mm.

To investigate the tunable strain distribution and auxetic behavior of the BMMI functional substrate, both finite element analysis (FEA) and tensile experiments were carried out. The results show that the heterogeneous strain distribution and the corresponding Poisson’s ratio can be effectively modulated by tailoring the modulus ratio between FS-1 (high modulus) and FS-2 (low modulus), in agreement with our design analysis. First, tensile simulations were performed on FS-1 alone to demonstrate the origin of its auxetic behavior, showing that the selected unit geometry undergoes longitudinal expansion under various lateral tensile strains, as presented in fig. S4. Second, functional substrates with five different modulus ratios between FS-1 and FS-2 (1:1, 6:1, 15:1, 52:1, and 144:1) were simulated under 5% strain to evaluate their strain modulation performance in Fig. 2C, which is directly related to the heterogeneous biosignal responses of the BMMI. With increasing modulus ratio, the heterogeneity of the strain distribution is amplified, resulting in smaller values of strain in the “low-strain” regions (FS-1) and larger strains in the “high-strain” regions (FS-2), which allow for tuning the sensors’ GF in both areas.

Furthermore, Poisson’s ratios of the five functional substrates, fabricated with varying PDMS modulus ratios, were characterized using a tensile tester, as shown in Fig. 2D. The progressive decrease in Poisson’s ratio (from 0.468 to −0.051) with increasing modulus ratio underscores the effective modulation enabled by the metamaterial backbone design while simultaneously enhancing the conformability of the BMMI to the skin compared to a flat substrate (fig. S5). In addition, all five functional substrates maintain stable mechanical performance (fig. S6), attributed to the strong interfacial bonding between the constituent materials, as detailed in Supplementary Text. Last, the photographs in Fig. 2E clearly illustrate the heterogeneous deformation of the BMMI, with smaller deformation in FS-1 and larger stretching in FS-2, under tensile strains ranging from 5 to 20%.

### Characterization of the BMMI

**Fig. 3.**
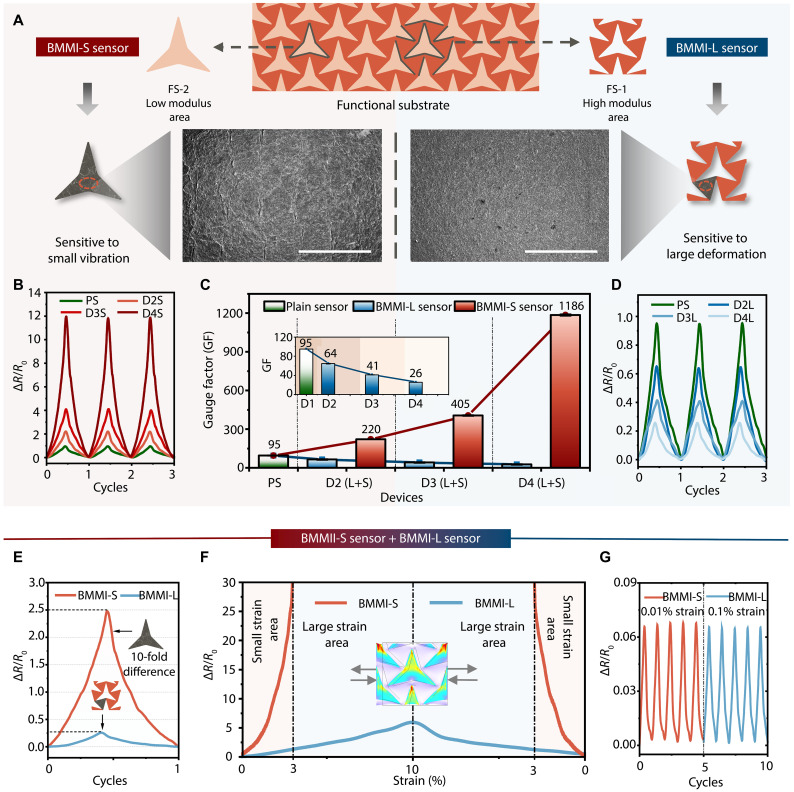
Characterization of the BMMI. (**A**) SEM images of the BMMI-S and BMMI-L sensors’ surfaces under 5% tensile strain highlight the differentiated crack formation resulting from strain engineering. Scale bars, 500 μm. (**B**) Relative resistance changes of BMMI-S sensors in PS, D2, D3, and D4, corresponding to BMMIs with modulus ratios of 1:1, 6:1, 15:1, and 52:1, respectively, under 1% cyclic stretching. (**C**) Comparison of GF between BMMI-S and BMMI-L sensors in PS, D2, D3, and D4. (**D**) Relative resistance changes of BMMI-L sensors in PS, D2, D3, and D4 under 1% cyclic stretching. (**E**) Relative resistance changes of BMMI-S and BMMI-L sensors in the BMMI with a modulus ratio of 15:1, showing a 10-fold difference. (**F**) Relative resistance changes of BMMI-S and BMMI-L sensors under 10% tensile strain and release. (**G**) Detection limit stability test for BMMI-S and BMMI-L sensors.

Integrated with graphene nanoplatelets (prepared by the high-pressure homogenization method characterized in figs. S7 and S8) on both FS-1 and FS-2, the heterogeneous strain distribution of the BMMI is reflected in the distinct crack densities of the graphene layers. Figure S9 presents the photograph of the fabricated BMMI, where FS-1 combined with the sensing layer forms the BMMI-S sensor, and FS-2 combined with the sensing layer forms the BMMI-L sensor. The scanning electron microscopy (SEM) images in Fig. 3A, scanned from the surfaces of the BMMI-S sensor (low modulus, sensitive to small vibrations) and the BMMI-L sensor (high modulus, responsive to large deformations) under 5% tensile strain, clearly reveal the differentiated crack formation resulting from strain engineering. Evidently, the strain-concentrated BMMI-S sensor exhibits longer, wider, and denser cracks compared to the strain-relaxed BMMI-L sensor. To facilitate visualization and comparison of the crack morphology, a quantitative analysis was conducted (fig. S10), further confirming that the crack density in the graphene layers governs the heterogeneous sensing responses of the BMMI.

In the previous subsection, the modulatory effect of the modulus ratio on the functional substrate was analyzed. Here, we quantitatively characterize how the modulus ratio regulates the strain-sensing capability of BMMI. All sensing signals are presented as relative resistance changes under various applied strain conditions, with the plain sensor (PS), D2, D3, and D4 corresponding to BMMIs fabricated with modulus ratios of 1:1, 6:1, 15:1, and 52:1, respectively, which exhibit stable and reproducible sensing outputs. The GF is calculated as (Δ*R*/*R*_0_)/ε, where ε denotes the applied strain. Figure 3B shows the performance of the BMMI-S sensors, where the relative resistance change gradually grows with the rising modulus ratio under identical cyclic stretching, indicating that the increasingly localized strain in BMMI-S boosts its sensitivity to small vibrations. Conversely, Fig. 3D presents the BMMI-L sensors’ performance, where the relative resistance change steadily declines with higher modulus ratios, suggesting that the more relaxed strain in BMMI-L effectively reduces minor noise interference during large deformations. As a result, the two opposite trends of the GF curves in Fig. 3C summarize how the modulus ratio modulates the BMMI’s performance. Compared to the PS, our designed BMMI achieves dual tunable GFs, with the GF of BMMI-S increasing by up to ~12 times and that of BMMI-L decreasing by about four times, thereby realizing heterogeneous sensing for small vibrations and large deformations with intrinsic capabilities for both signal enhancement and noise suppression.

Taking advantage of its dual tunability, the BMMI can be customized to suit specific application scenarios. In this work, we selected D3 (modulus ratio of 15:1) for the detection of diverse biomechanical signals on the neck, as it provides an appropriate GF difference, with the GFs of BMMI-S and BMMI-L reaching 41 and 405, respectively. As demonstrated by the relative resistance changes in Fig. 3 (E and F), the BMMI achieves a 10-fold difference in GF within a single functional substrate, with BMMI-S excelling in small-strain regions and BMMI-L performing well for large-strain regions. Both BMMI sensors demonstrate consistent and stable responses under cyclic stretching with varying strain amplitudes (figs. S11 and S12), as well as outstanding durability over 10,000 cycles (figs. S13 and S14). In addition, BMMI-S and BMMI-L display distinct detection limits, with BMMI-S capable of sensing strains as small as 0.01%, while BMMI-L detects strains down to 0.1% (Fig. 3G). Table S1 compares the performance of the BMMI with representative wearable interfaces.

### Detection of heterogeneous MDA

To assess the superior capabilities of the BMMI in capturing spatially heterogeneous signals on the skin surface under real-world conditions, two BMMI-S and two BMMI-L sensors were systematically distributed across four metamaterial units corresponding to the neck region for the simultaneous acquisition of multiple adjacent physiological signals. When the user wears the four-channel BMMI, a broad spectrum of mechanical deformations, including small vibrations generated by the carotid artery and throat region, and large deformations from the lateral neck muscles can be selectively captured by BMMI-S and BMMI-L, respectively.

**Fig. 4.**
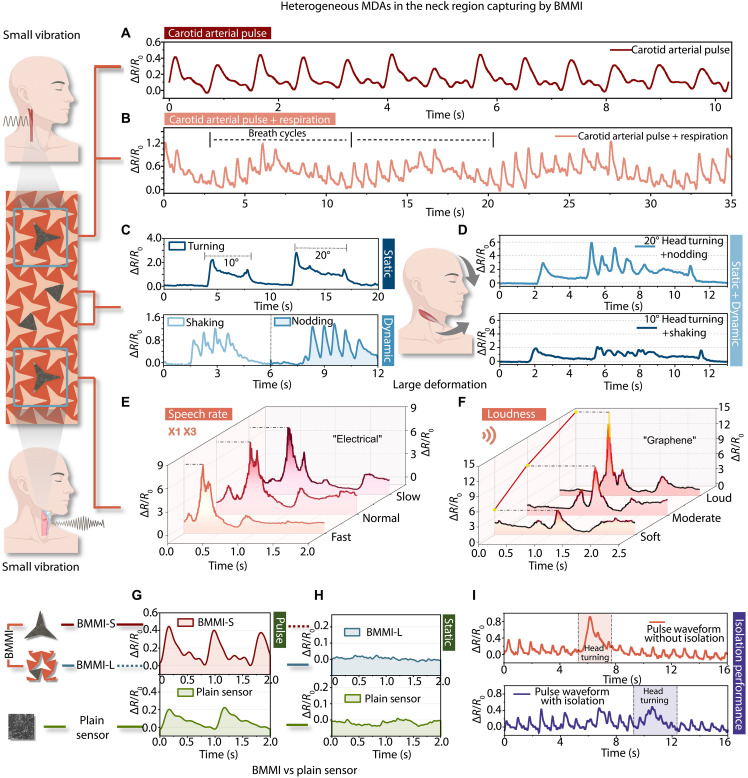
Detection of heterogeneous MDAs. (**A**) Carotid arterial pulse waveform captured by the BMMI-S sensor. (**B**) Respiratory cyclic waveform superimposed on the pulse signal captured by the BMMI-S sensor. (**C**) Static actions, such as turning the head 10° or 20° left, and dynamic movements like nodding and shaking, detected by the BMMI-L sensor. (**D**) Composite signals generated by nodding or shaking the head while it is held at a 20° or 10° leftward rotation, detected by the BMMI-L sensor. (**E**) Throat vibration signals of “Electrical” at fast, normal, and slow speech rates, obtained by the BMMI-S sensor. (**F**) Throat vibration signals of “Graphene” at soft, moderate, and loud volumes, obtained by the BMMI-S sensor. (**G**) Comparison of pulse signals collected by the BMMI-S and PSs. (**H**) Comparison of static signals collected by the BMMI-L and PSs. (**I**) Comparison of pulse signals during head turning, collected by the BMMI-S sensors with and without isolation frames.

Specifically, the BMMI-S sensor reliably detects subtle pulse signals with regular intervals in the carotid artery region (Fig. 4A). In addition, owing to the unique anatomical characteristics of the neck, the BMMI-S simultaneously captures the respiratory cyclic waveform superimposed on the pulse signal (Fig. 4B), enabling concurrent assessment of both cardiovascular and respiratory activities. Meanwhile, the central BMMI-L sensors capture a variety of head movements, encompassing both static and dynamic motions. As shown in Fig. 4C, static actions, such as turning the head left by 10° or 20°, and dynamic movements like nodding and shaking the head are distinctly differentiated and recorded. Notably, the BMMI-L sensor also resolves compound mechanical events. For instance, Fig. 4D presents the composite signal generated by nodding or shaking the head while it is held at a 20° or 10° leftward rotation. Last, another BMMI-S sensor placed over the laryngeal region precisely discerns variations in spoken words, speech rates, and loudness levels. Two representative words, “Electrical” and “Graphene,” are evaluated under three different speaking rates and three loudness levels. As illustrated in Fig. 4 (E and F), although the same word maintains consistent waveform features across varying conditions, the signals exhibit distinct scaling in both temporal duration and amplitude, underscoring the substantial sensitivity of the BMMI-S to subtle vocal vibrations.

The four-channel BMMI successfully acquired high-fidelity and high-quality multimodal biosignals from the neck region. This performance benefits from spatially targeted sensing, which amplifies signal magnitude and effectively suppresses unwanted noise, thereby establishing a robust foundation for reliable cognitive and affective analyses based on diverse physiological information. To more intuitively demonstrate the advantages of heterogeneous sensing, comparative experiments were conducted using PSs. In the context of arterial pulse detection, enhanced sensor sensitivity is critical for precise monitoring, which underpins effective physiological analysis (*38*). When using the BMMI-S sensor with a GF as high as 405, the acquired pulse waveforms (Fig. 4G) exhibit an idealized pattern with three gradually weakening positive peaks. These features enable detailed extraction of arterial pulse dynamics (*38*). In contrast, the PS produced signals with approximately half the amplitude and failed to preserve many critical waveform details. Moreover, the BMMI-L sensor demonstrated excellent resistance to motion artifacts under static conditions, as evidenced by a comparison with a PS placed on the lateral neck during head rest (Fig. 4H), with quantitative analysis provided in fig. S15. Meanwhile, the BMMI demonstrates superior signal stability under prolonged wear and repeated attachment and detachment, as shown in figs. S16 and S17. These findings collectively highlight the exceptional capability of the BMMI system in the broad detection of heterogeneous MDA.

With the aim of reducing cross-talk between the BMMI-S and BMMI-L sensors, polyimide (PI) isolation frames were incorporated around the two BMMI-S sensors. The FEA results shown in fig. S18 indicate that when tensile strain is applied from both ends, the strain within the isolated region enclosed by the frames is substantially reduced. At the same time, high sensitivity is maintained inside the frame. This finding is consistent with the results presented in Fig. 4I, where cross-talk from head rotation was substantially mitigated, effectively preserving the integrity of the pulse waveform. Quantitative analysis supporting this finding is provided in fig. S19.

### Multimodal communication analysis

Humans rely on verbal and nonverbal communication to convey information comprehensively in daily life. Multimodal communication analysis plays a crucial role in understanding and enhancing human interaction by interpreting various forms of communication. Here, we used our wireless BMMI (fig. S20) in conjunction with a bespoke deep learning model, CA-Net, to perform multimodal communication analysis for inferring internal cognitive-affective states, including emotion, attitudes, and attention.

**Fig. 5.**
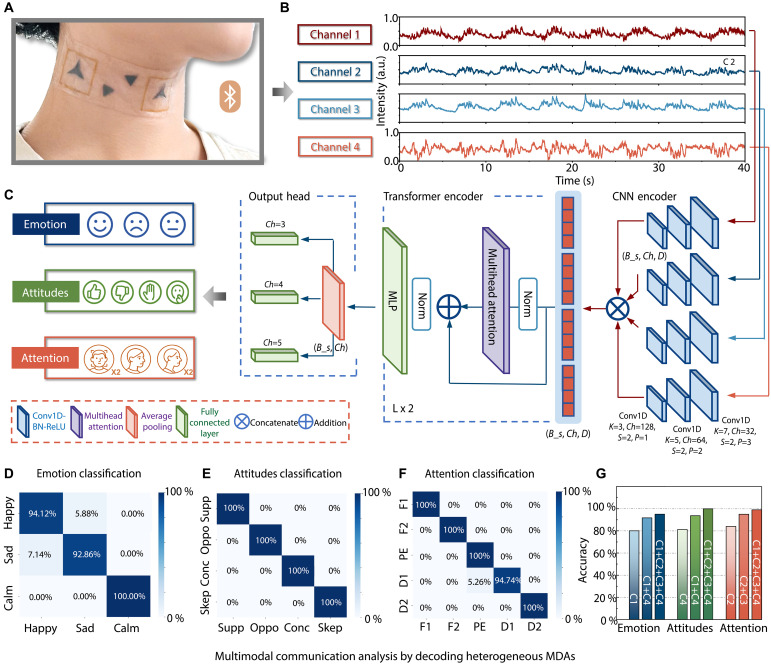
Multimodal communication analysis by decoding heterogeneous MDAs. (**A**) Photograph of the user wearing the four-channel wireless BMMI. (**B**) Four-channel signals collected by the wireless BMMI: channel 1, subtle carotid pulse and respiratory activity; channels 2 and 3, static and dynamic head movements; channel 4, laryngeal vibrations. a.u., arbitrary units. (**C**) The structure of the CN-Net model, based on a hybrid architecture encoder and three parallel output heads, enables precise classification of emotional, attitudinal, and attentional states. (**D** to **F**) Confusion matrices for the classification of three emotions, four attitudes, and five levels of attention. (**G**) Accuracy comparison of ablation studies conducted for the three internal states.

Cognition and affect can be decoded from a range of high-fidelity physiological and behavioral signals, such as pulse, respiration, speech, and muscle movement (*39*–*42*). Given the complex interplay among diverse biosignals that contribute to inherently multidimensional internal states, targeted multimodal perception is essential for robust and accurate recognition. Leveraging its unique ability to detect heterogeneous MDA, the four-channel BMMI can simultaneously capture multiple biophysical signals via BMMI-S and BMMI-L sensors placed at different regions of the neck, each exhibiting distinct strengths and characteristics (Fig. 5, A and B). These valuable signals contain substantial spatiotemporal details hidden in physiological activities, which can be used for comprehensive analysis of the human cognitive and affective states.

On the basis of powerful data-driven deep learning techniques, we propose a model, CA-Net (Fig. 5C), which incorporates a hybrid architecture encoder and three parallel output heads for precise classification of emotional, attitudinal, and attentional states. This approach, which fully considers the spatiotemporal diversity of the four-channel sequential signals—channel 1 for detecting subtle carotid pulse and respiratory activity, channels 2 and 3 for capturing static and dynamic head movements, and channel 4 for recording laryngeal vibrations—adopts a hybrid backbone that integrates convolutional neural networks (CNNs) (*43*) and transformers (*44*), thereby enabling cascaded feature extraction and cross-channel fusion.

Specifically, our CNN encoder features a quad-branch architecture, where each branch comprises three convolutional layers, each of them followed by a BatchNorm layer and a ReLU (rectified linear unit) layer, enabling efficient local feature extraction from each channel through the strong inductive bias of convolution inspired by PESNet (*45*). In addition, the hierarchical outputs shorten the sequence length, thereby reducing computational overhead for the subsequent transformer encoder (*46*). The four resulting feature sequences are then concatenated along the hidden dimension (*D*) and fed into the transformer to establish long-range dependencies. For the transformer stage, the model omits the original positional embedding process for each feature sequence to avoid the order of input channels on the consistency of prediction. Accordingly, the transformer encoder is designed with two layers, each containing a multihead self-attention head, which uses its global receptive field and consistent resolution to establish global interactions among all feature tokens from the four signal channels. Last, an average pooling layer condensed the hidden dimension (*D*) to feed the fused features into parallel classification heads for specific tasks.

A total of 1200 sets of four-channel data were collected from five subjects, encompassing three emotional states (happy, sad, and calm), four attitudes (support, oppose, concern, and skeptical), and five levels of attentional engagement (focused ×2, partially engaged, and distracted ×2), with 100 sets for each category (figs. S21 to S23). Of the total dataset, 70% was used for training the model, while 20 and 10% were allocated for validation and testing, respectively. As shown by the loss curves in figs. S24 to S26, our proposed CA-Net exhibits fast convergence during training and maintains consistent performance during testing. As a result, it achieved classification accuracies of 95% for emotion, 100% for attitude, and 99% for attention recognition. The corresponding confusion matrices are displayed in Fig. 5 (D to F). To further validate the effectiveness of CA-Net in cognition and affect recognition, we conducted comparative experiments against conventional recurrent neural network– and pure transformer-based models, as presented in table S2. CA-Net consistently outperformed these models in classification accuracy, showcasing its outstanding ability to capture spatiotemporal patterns across multimodal biosignals. Moreover, fig. S27 demonstrates effective generalization of CA-Net to new users. Table S3 provides a comparative overview of representative wearable sensor–based approaches for affective and cognitive state recognition, which contextualizes the performance of the proposed method.

The key advantage and characteristics of multimodal integration were assessed through an ablation study in which individual channels were selectively used, or the number of input signals was reduced, to determine the contribution of the four-channel data to the overall classification performance. For example, in emotion recognition, using only the single-channel data from channel 1 at the carotid artery, following conventional approaches, resulted in an accuracy of just 80%, notably lower than the 95% achieved with the multimodal setup. Moreover, when heterogeneous MDA was excluded and only the small vibration signals from channels 1 and 4 were used, the accuracy dropped to 91.67%, indicating that critical affect-related features were not fully captured. As shown in Fig. 5G, similar ablation studies were conducted for the other two internal states, yielding consistent trends, with the corresponding confusion matrices presented in figs. S28 to S30. These results demonstrate that although classification remains feasible with single-modality input, the absence of multimodal synergy leads to substantial degradation in performance. Therefore, by simultaneously acquiring spatially and temporally distributed heterogeneous MDAs using our BMMI system, we fully leveraged the synergistic effect of multimodal integration to decode and recognize complex internal states through the bespoke CA-Net model, achieving consistently higher classification accuracy across all categories.

## DISCUSSION

By mimicking both the surface morphology and tactile functionality of human skin, we have introduced a BMMI device that enables high-precision detection of spatiotemporally distributed heterogeneous MDA. Applied to a conformable artificial skin device, this capability allows for targeted capture of adjacent skin deformations associated with a range of physiological information of crucial importance for a deeper understanding of human behavior and nonverbal communication. Characterized by its unique auxetic metamaterial backbone designed for strain engineering, the BMMI goes beyond any existing concepts of flexible human-machine interface and achieves hardware-level signal amplification and noise suppression on the same functional substrate, with application-specific tunable performance. As a multimodal communication interface, on the basis of its high-fidelity multichannel biophysical signals and the CA-Net model, the wireless BMMI system achieves unprecedented performance in the automatic analysis and classification of cognitive and affective states. The ablation studies conducted here provide valuable insights in the importance of the synergistic effect of heterogeneous biophysical signals associated with MDA. Building upon the technology platform introduced in this study to decode heterogeneous MDA, future efforts could extend the approach with further adjustments to the modulus ratio, exploring other geometric configurations and distributions of sensing materials, opening promising avenues for health diagnosis, natural human-machine interaction, and beyond.

## MATERIALS AND METHODS

### Materials

The Dowsil SYLGARD 184 Silicone Elastomer Kit (comprising a prepolymer and curing agent) was sourced from Conro Electronics Ltd. and used for fabricating FS-1 and FS-2 of the functional substrate. Ecoflex 00-10 (Part A and Part B) was obtained from Bentley Advanced Materials for the fabrication of FS-3. TIMREX KS 25 Graphite (synthetic graphite, 25-μm particle size) was provided by IMERYS. Sodium deoxycholate (SDC; ≥97%) and sodium carboxymethyl cellulose (CMC-Na, average molecular weight of 700,000), used as the surfactant and binder for ink preparation, were both purchased from Sigma-Aldrich. PI Kapton tape (0.1-mm thickness) was sourced from AliExpress. The three-dimensionally printed (3D-printed) molds used for substrate fabrication were supplied by Protolabs Ltd. Stretchable printable silver paste (DM-SIP-2006) for electrode fabrication was purchased from Dycotec Materials Ltd.

### Fabrication of the BMMI

The fabrication of the BMMI involves the following steps using a 3D-printed mold designed in the form of an auxetic metamaterial with star-shaped perforations, along with a patterned mask. For the FS-1 of the functional substrate, a release agent was sprayed over the surface of the 3D-printed mold and left to dry naturally at room temperature. The PI frame was placed inside the mold. O_2_ plasma treatment was applied to increase the bonding between PI and PDMS. The high-modulus PDMS (prepolymer:curing agent = 10:1) was cast into the auxetic metamaterial mold and cured in an oven at 150°C for 23 min. For the FS-3 of the functional substrate, Ecoflex 00-10 Part A and Part B were mixed in a 1:1 ratio, followed by degassing of the mixture in a vacuum oven. Ecoflex 00-10 was spin-coated on FS-1 at 600 rpm for 180 s (about 100 μm thick) and then cured at 80°C for 30 min. For the FS-2 of the functional substrate, FS-1, PI, and FS-3 were peeled off from the mold. The low-modulus PDMS (with prepolymer–to–curing agent weight ratios of 10:1, 20:1, 30:1, 40:1, and 50:1 for PS, D2, D3, D4, and D5, respectively) was poured into the star-shaped perforations of FS-1 and then cured in an oven at 65°C for 24 hours. For the sensing layer, both FS-1 and FS-2 were treated with O_2_ plasma to generate silanol (Si−OH) terminal groups on their surfaces. The patterned mask was placed onto the functional substrate on a hot plate set to 100°C, and then graphene ink (50 g/liter) was sprayed at 137.9 kPa from a height of 23 cm for 2 min. Electrodes were fabricated using stretchable silver paste (cure in an oven at 80°C for 40 min) and copper wire.

### Fabrication of graphene nanoplatelet ink

The graphene nanoplatelets that constitute the sensing layer are prepared using a high-pressure homogenizer according to the steps below. An SDC solution was prepared in deionized water at a concentration of 2.5 g/liter to inhibit filler aggregation via electrostatic repulsion. TIMREX KS 25 graphite flakes were added to the SDC solution at 50 g/liter, and the mixture was blended using a dissolver at 500 rpm for 30 min. The graphite was exfoliated by processing the mixture through a high-pressure homogenizer (PSI-40) equipped with a dual-slot deagglomeration chamber (87 μm), applying a pressure of 700 bars for 70 cycles. CMC-Na was added to the resulting graphene dispersion at 2.5 g/liter as a binder to enhance flake stability. The ink was stirred thoroughly at room temperature for 3 hours to ensure complete dissolution of CMC-Na.

### Modulation of PDMS Young’s modulus

PDMS, a synthetic silicone polymer, is widely used in biomaterials research. Its Young’s modulus can be tuned by adjusting the cross-linker concentration, curing time, or curing temperature (*47*). In this study, we fabricated PDMS layers with Young’s moduli ranging from 0.018 to 2.59 MPa using the Dowsil SYLGARD 184 Silicone Elastomer Kit. Specifically, the high-modulus FS-1 layer, with Young’s modulus of 2.59 MPa, was prepared by mixing the prepolymer and curing agent at a 1:1 weight ratio, followed by curing for 23 min at 150°C (*48*). By varying the weight ratio of prepolymer to curing agent from 20:1, 30:1, and 40:1 to 50:1, the low-modulus FS-2 layers achieved Young’s moduli of 0.445, 0.17, 0.05, and 0.018 MPa, respectively, after curing at 65°C for 24 hours (*47*). As a result, five functional substrates with modulus ratios of 1:1, 6:1, 15:1, 52:1, and 144:1 were fabricated for comparative analysis.

### Finite element analysis (FEA)

The strain distribution and deformation of the functional substrate for BMMI were analyzed using the commercial FEA software COMSOL Multiphysics 6.2. Considering that PDMS exhibits linear elastic behavior under strains of up to 20%, a linear elastic material model was adopted in the simulations (*49*, *50*). The Young’s moduli were set to 2.59 MPa for the high-modulus FS-1 and to 0.445, 0.17, 0.05, and 0.018 MPa for the low-modulus FS-2, corresponding to functional substrates with modulus ratios of 1:1, 6:1, 15:1, 52:1, and 144:1, respectively. The Poisson’s ratio of PDMS was set to 0.49. A solid mechanics (solid) interface was used, and a stationary study was performed to simulate deformation. Uniaxial tensile strain was modeled by applying fixed constraints to one end of the substrate and a prescribed displacement to the opposite end along the longitudinal direction. The computational domain was discretized using a physics-controlled mesh with a finer element size.

### Characterization of the BMMI

The graphene flakes were characterized using atomic force microscopy (Bruker Icon). The morphology of the BMMI was examined with SEM (Magellan 400), with a gold layer sputter-deposited onto the samples before measurement. The strain-sensing performance of the BMMI-S and BMMI-L sensors was tested using the INSTRON universal tensile testing system. The mechanical properties of the functional substrates were assessed with the Hounsfield Universal Testing Machine (max. 1 kN). The resistance response during cyclic stretching and release, as well as the detection of heterogeneous MDAs, was captured using PalmSens Potentiostats and the designed four-channel wireless readout module, both with a sampling rate of 500 Hz.

### Crack analysis based on SEM images

The SEM images of the sensing layer were enhanced to facilitate the visualization and analysis of cracks. All SEM image processing, crack detection, and quantitative analysis were conducted using MATLAB R2021b with the Image Processing Toolbox. The detailed processing workflow is as follows. First, the original RGB (red, green, and blue) images were converted to grayscale and enhanced using adaptive histogram equalization with Rayleigh distribution and a clip limit of 0.01. The resulting images were normalized and mapped with a jet color map to improve contrast and facilitate visual inspection of potential crack regions. Second, crack skeleton images were generated through a multistep procedure: Laplacian-of-Gaussian filtering was applied to the grayscale images to highlight fine crack-like structures, followed by thresholding and morphological operations to clean and connect crack segments. The resulting binary crack maps were skeletonized and slightly dilated to improve visibility. These final binary images, highlighting the extracted crack skeletons, were subsequently used for quantitative crack analysis, including total length and density calculations. The results are shown in fig. S10.

### Wireless readout module of BMMI

To enable portable and real-time data acquisition, a compact wireless readout module was integrated into the BMMI device. The system is built around an ESP32 microcontroller, which provides both high-speed data handling and BLE (Bluetooth Low Energy) wireless transmission capabilities. One analog-to-digital converter channel of the ESP32 is connected to a one-to-four analog multiplexer, enabling time-multiplexed sampling of four analog signal channels at 500 Hz per channel using a dedicated analog front-end. The modular architecture allows for straightforward expansion to additional input channels, accommodating higher channel-count applications. The acquired data are transmitted wirelessly to a nearby receiver (e.g., laptop or mobile device), enabling untethered operation and remote monitoring. The readout module is powered by a 3.7-V, 350-mA·hour lithium polymer battery.

### Data collection from human subjects

The experiment involving human subjects was approved by the Ethics Committee of the Department of Engineering at the University of Cambridge (no. 566). All participants were provided with a Participant Information Sheet and asked to complete and sign a Participant Consent Form before their participation in the experiment. We provide a detailed protocol to collect high-quality, labeled biosignal data that can be used to classify human emotions, attitudes, and attention states. The following experimental setup was established: participants: a group of five healthy students from the University of Cambridge participated (average age, 26; two females and three males); environment: controlled lighting and minimal noise, comfortable seating, and computers with high-resolution displays and an audio system; stimuli: a set of visual, auditory, and video stimuli designed to elicit a range of emotions (happy, sad, and calm), attitudes (support, oppose, concern, and skeptical), and attention (focused, partially engaged, and distracted); data acquisition: participants wore a wireless four-channel BMMI and were instructed to watch emotion-eliciting video clips, answer the survey questions with four different attitudes, and direct their attention either to a primary screen or to distracting stimuli presented on a secondary screen; summary of data collection: a total of 1200 datasets were collected, including three emotional states (happy, sad, and calm), four attitudes (supportive, opposing, concerned, and skeptical), and five levels of attentional engagement (focused ×2, partially engaged, and distracted ×2), with 100 sets per category and 2500 data points per set.

### CA-Net deep learning–based model

CA-Net, a deep learning–based model designed for multimodal communication analysis, incorporates a hybrid architecture encoder and three parallel output heads for precise classification of emotional, attitudinal, and attentional states. The sequence length of each input is unified as 2500 data points. *B_s*, *Ch*, and *D* denote the batch size, the number of channels, and hidden dimension, respectively. For the CNN encoder, (*B_s*, *Ch*, *D*) = (16, 128, 312). For the transformer encoder, (*B_s*, *Ch*, *D*) = (16, 128, 1248). For the average pooling layer, (*B_s*, *Ch*) = (16, 128). During the training process, we use the cross-entropy loss to constrain our model and Adam optimizer with a learning rate of 0.001. All comparison and ablation studies were trained with a batch size of 16 for 20 epochs. The average throughput of CA-Net is 324.87 ± 13.85 samples/s.

## Supplementary Materials

### The PDF file includes:

Supplementary Text Figs. S1 to S30 Tables S1 to S3 Legends for movies S1 to S3 References
