# Supplementary material for: Biomimetic metamaterial–based interface for decoding heterogeneous mechanodermal activity
Source: Sci Adv. 2026 Apr 22;12(17):eaee2625. doi: 10.1126/sciadv.aee2625 (PMC13101874; doi:10.1126/sciadv.aee2625)
Supplement: Supplementary file 1 — Supplementary Text Figs. S1 to S30 Tables S1 to S3 Legends for movies S1 to S3 References [file sciadv.aee2625_sm.pdf]

Science Advances
AAAS

Supplementary Materials for

Biomimetic metamaterial–based interface for decoding heterogeneous mechanodermal activity

Muzi Xu *et al.*

Corresponding author: Luigi G. Occhipinti, L.G.Occhipinti@lboro.ac.uk

Sci. Adv. **12**, eaee2625 (2026)
DOI: 10.1126/sciadv.aee2625

The PDF file includes:

Supplementary Text
Figs. S1 to S30
Tables S1 to S3
Legends for movies S1 to S3
References

Other Supplementary Material for this manuscript includes the following:

Movies S1 to S3

Supplementary Text

Interfacial bonding between constituent materials

The BMMI comprises a functional substrate and a sensing layer made of different materials. Numerous studies have explored the interfacial bonding between PDMS of varying moduli and other materials like Ecoflex, graphene, and polyimide *(51–56)*.

As indicated by previous studies *(51, 52)*, PDMS with varying moduli, as well as PDMS and Ecoflex, exhibit strong interfacial bonding regardless of the curing sequence, resulting in a substrate that meets the requirements of our application. Furthermore, by introducing silanol (Si−OH) terminal groups through oxygen plasma treatment, both FS-1 (high-modulus PDMS) and FS-2 (low-modulus PDMS) exhibit good interfacial bonding with the sensing layer (graphene nanoplatelets) and the polyimide film *(53–56)*. The stable force–strain curves in mechanical testing (fig. S6) indicate strong interfacial bonding between constituent materials, laying the foundation for the outstanding signal stability and durability of our BMMI.

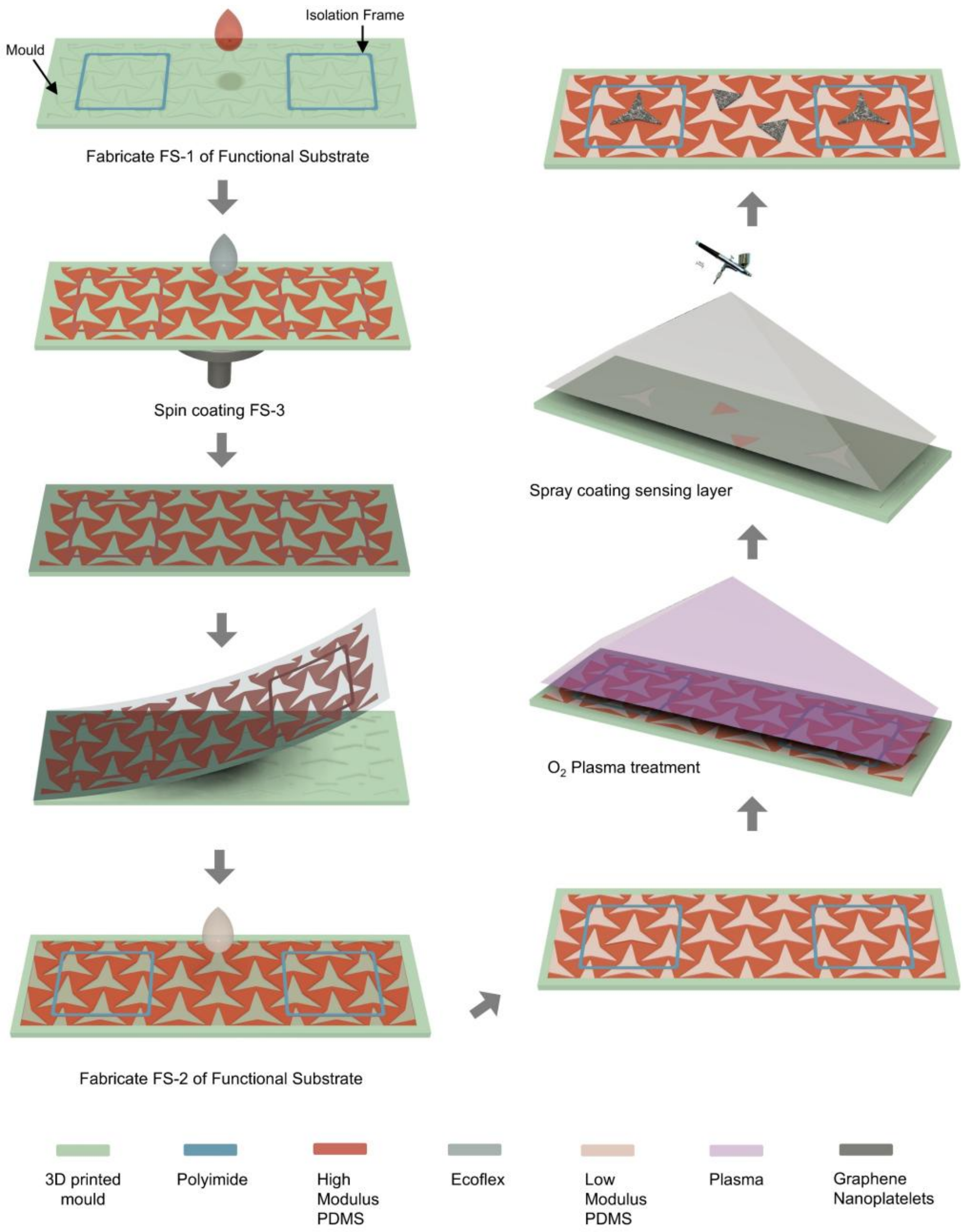

Fig. S1. Schematic of the fabrication process of the Biomimetic Metamaterial-based Interface (BMMI). Using a 3D printed mold designed in the form of an auxetic metamaterial with star-shaped perforations, the three components of the functional substrate were fabricated in the sequence of FS-1, FS-3, and FS-2. Subsequently, with the patterned mask, BMMI-S and BMMI-L were completed.

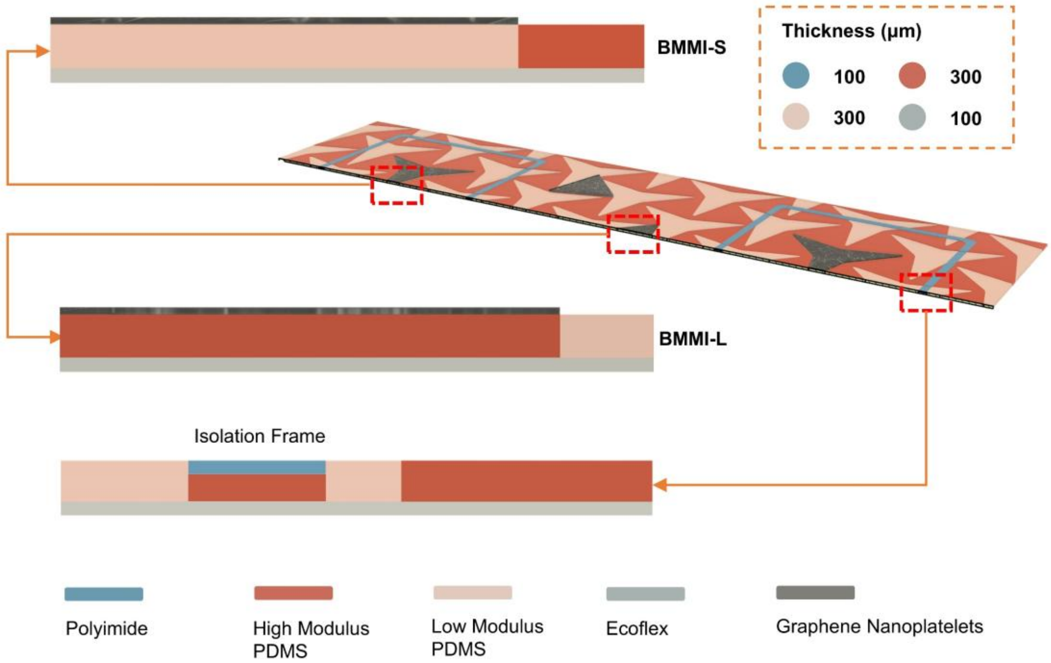

Fig. S2. Cross-sectional structure and thickness of Biomimetic Metamaterial-based Interface (BMMI). The total thickness of the BMMI is 400μm, with FS-1 (high modulus) and FS-2 (low modulus) comprising 300μm, while FS-3 (Ecoflex) and the isolation frame each account for 100μm.

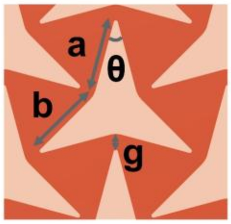

Auxetic Metamaterial Parameters

**θ:** Hinging angle	**θ:** 30°
**a:** Length of the sides	**a:** 8mm
**b:** Length of the sides	**b:** 8mm
**r=a/b :** the ratio between the side lengths	**r=a/b :** 1
**g:** Distance between each star-shaped region	**g:** 1.5mm

Fig. S3. Dimensions of the BMMI Metamaterial Unit. Multiple metamaterial units, formed by equilateral triangles with a 30° hinging angle, together constitute the complete BMMI functional substrate.

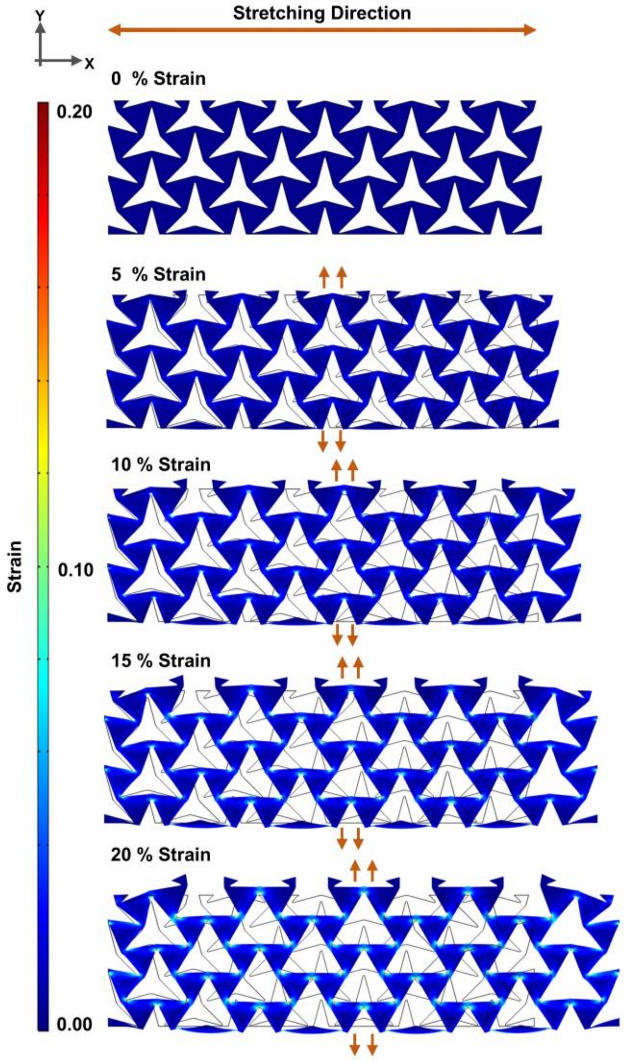

Fig. S4. The FEA results on the auxetic behavior of FS-1 (high modulus) under different strains. This selected geometry undergoes longitudinal expansion under various lateral tensile strains, forming the basis for the modulation of the BMMI.

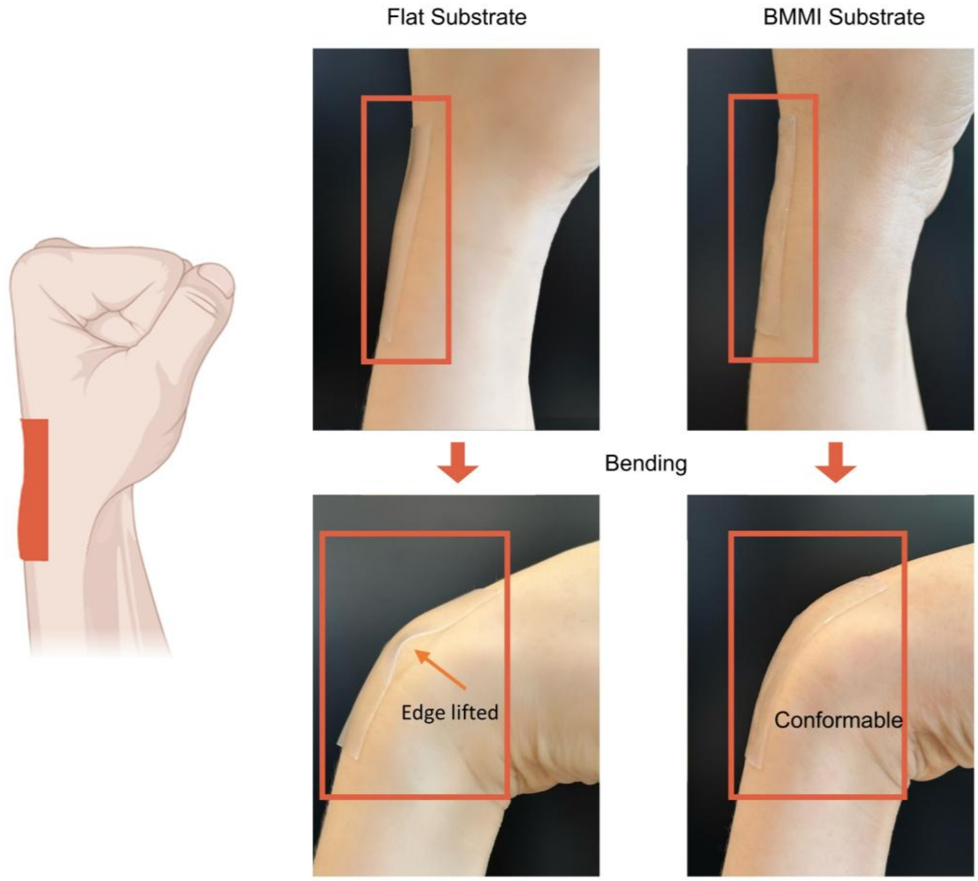

Fig. S5. Conformability test of the BMMI compared to a flat substrate. Both the flat substrate and the BMMI functional substrate were attached to the wrist. When bending, the edge of the flat substrate lifted, whereas the BMMI substrate remained closely adhered during bending. Some elements were created using BioRender.com.

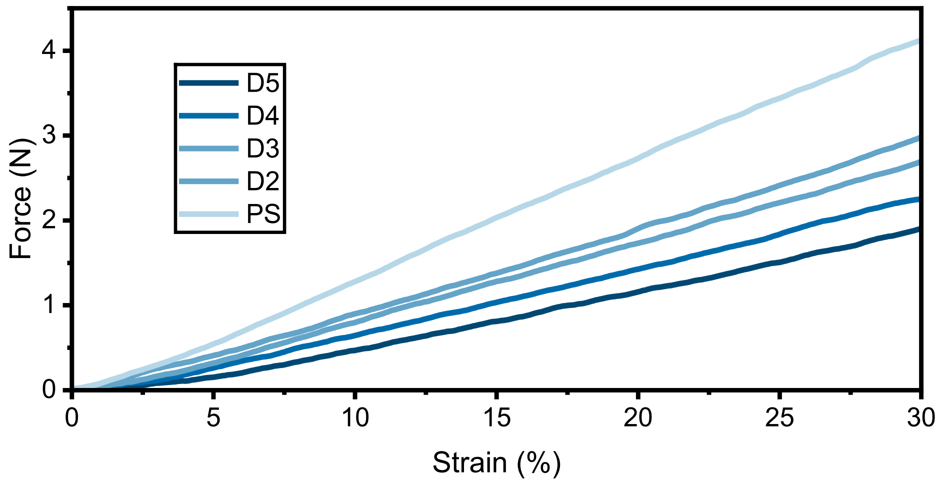

Fig. S6. Tensile force–strain curves of the functional substrates fabricated with different modulus ratios between the high-modulus FS-1 and the low-modulus FS-2. PS, D2, D3, D4, and D5 represent functional substrates with modulus ratios of 1:1, 5:1, 15:1, 50:1, and 150:1, respectively.

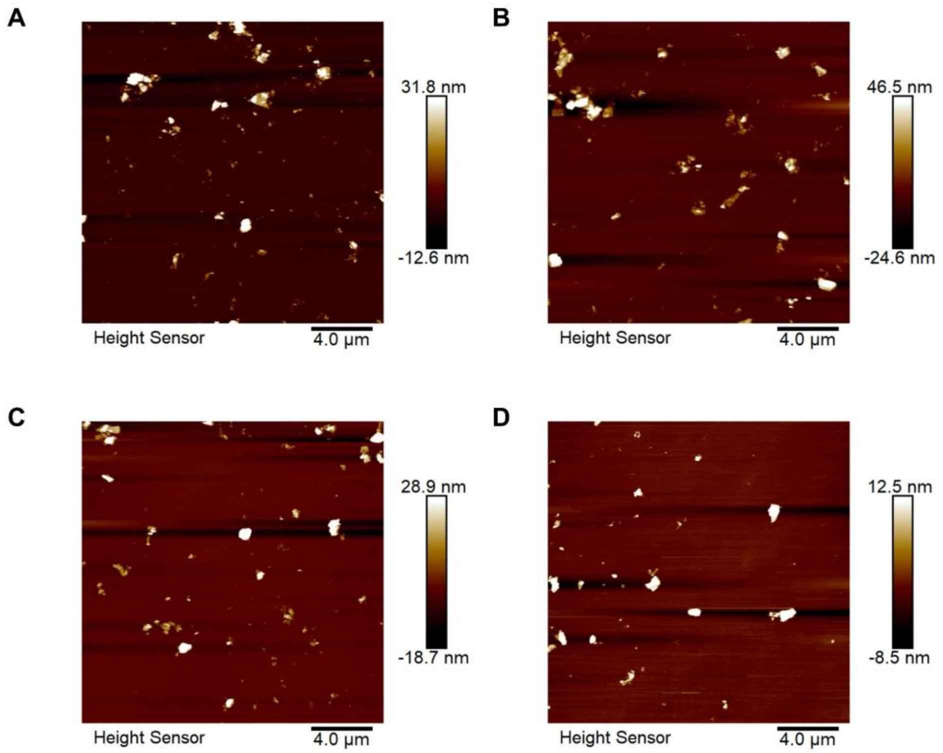

Fig. S7. AFM images (20 µm × 20 µm) of graphene nanoplatelets, prepared by High-Pressure Homogenization (HPH), were scanned at four distinct locations.

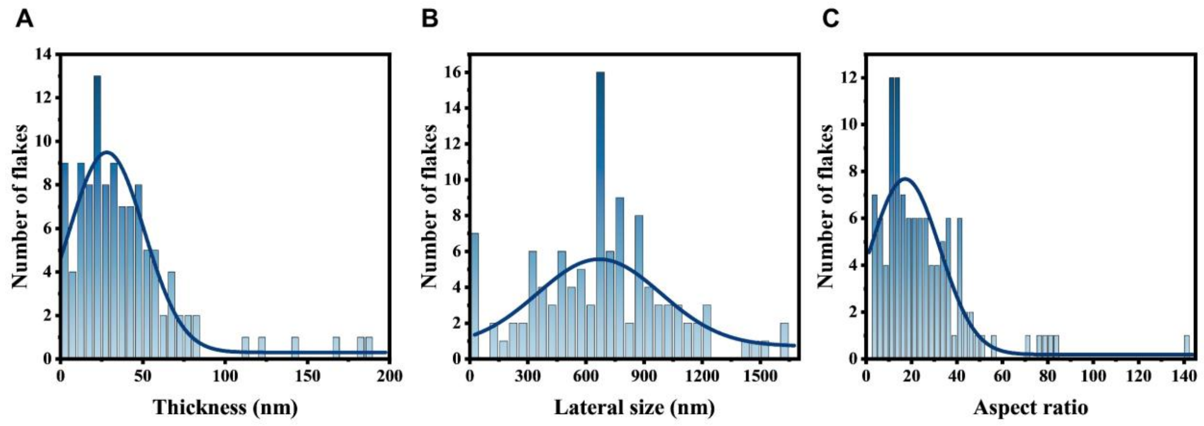

Fig. S8. Characterization of graphene nanoplatelets fabricated by High-Pressure Homogenizer (HPH). (A-C) The thickness, lateral size, and aspect ratio distributions of graphene flakes, derived from a sample of 100 randomly selected flakes across four AFM images shown in fig. S9.

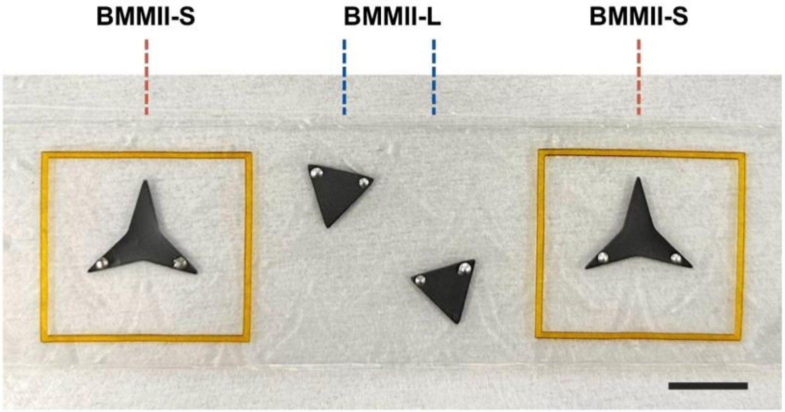

Fig. S9. The photograph of the BMMI. Scale bar 1cm.

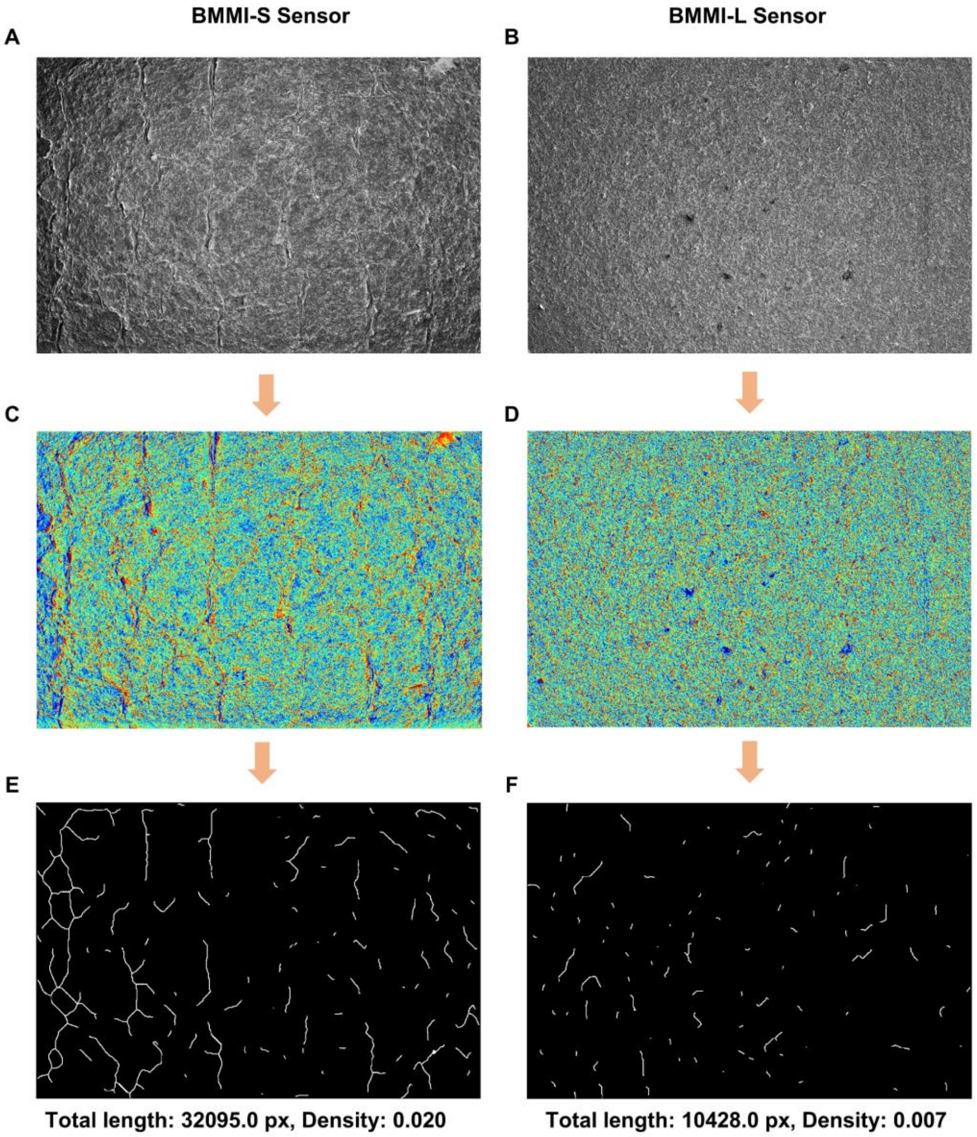

Fig. S10. Crack Analysis Based on SEM Images of BMMI-S and BMMI-L. (A-B) SEM images of BMMI-S and BMMI-L under 5% strain, reused from Fig. 3A. (C-D) SEM images of BMMI-S and BMMI-L after adaptive histogram equalization for contrast enhancement. (E-F) Corresponding crack skeleton images used for quantitative analysis, including measurements of total crack length and crack density for BMMI-S and BMMI-L, respectively.

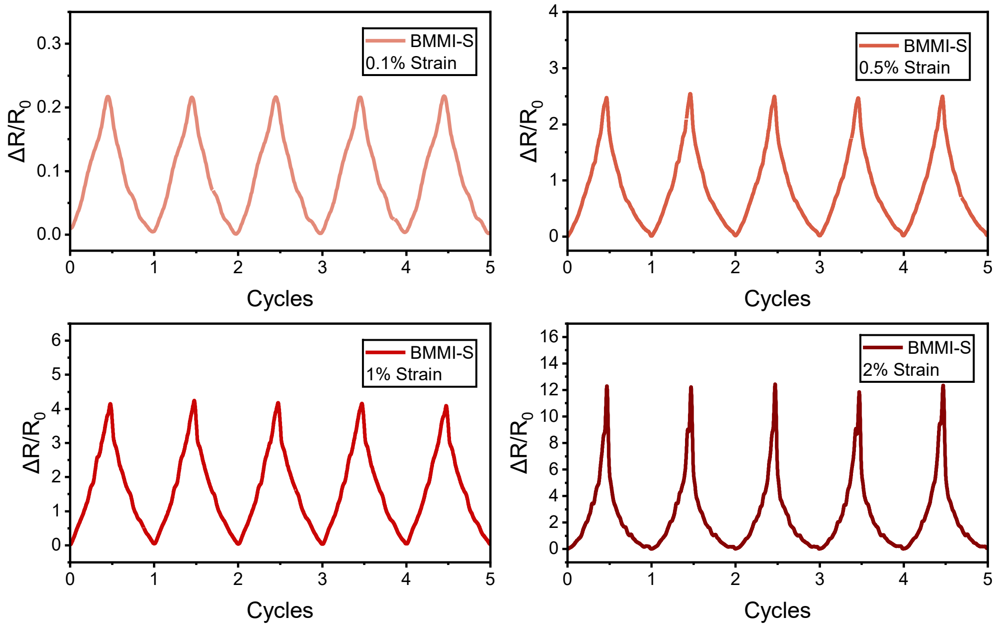

Fig. S11. Cyclic stability tests of BMMI-S under tensile strains of 0.1%, 0.5%, 1%, and 2%.

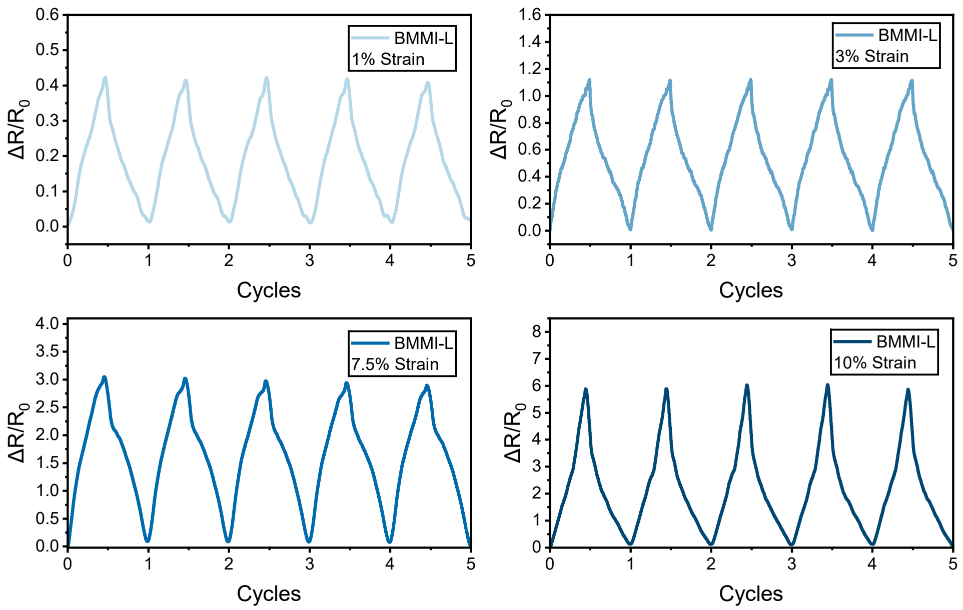

Fig. S12. Cyclic stability tests of BMMI-L under tensile strains of 1%, 3%, 7.5%, and 10%.

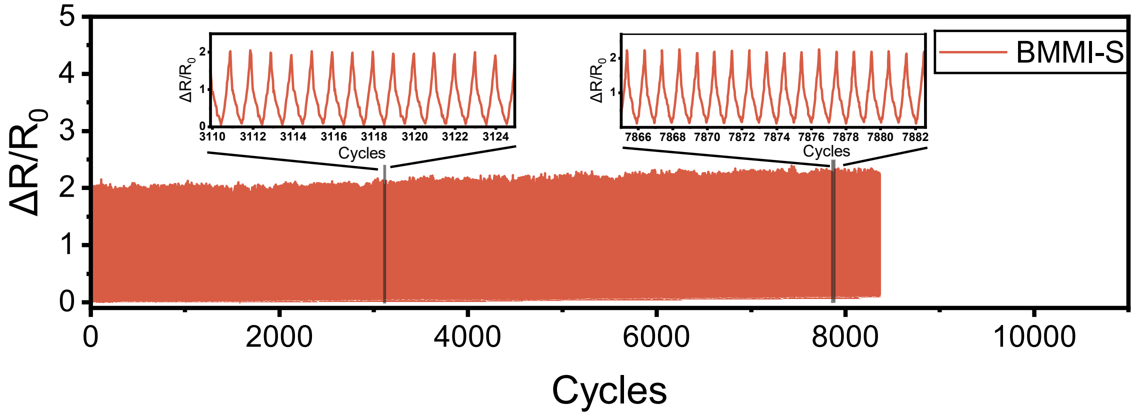

Fig. S13. Durability test of BMMI-S by multicyclic stretching and releasing over 10,000 cycles under 0.5% strain.

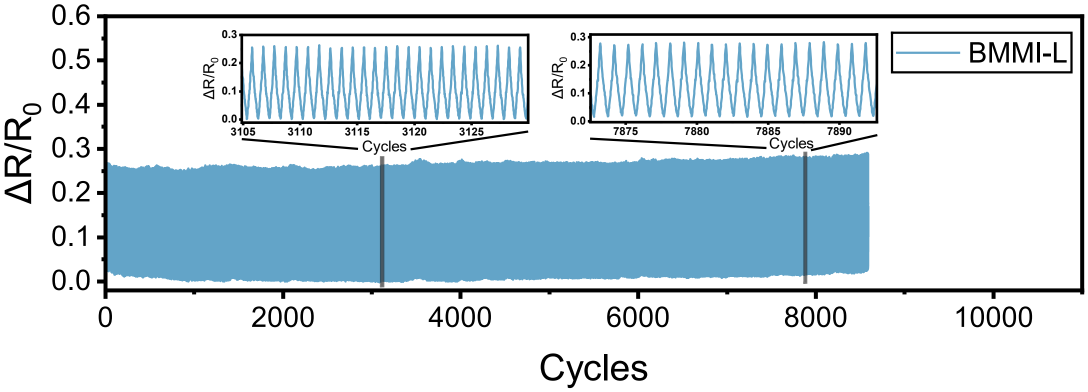

Fig. S14. Durability test of BMMI-L by multicyclic stretching and releasing over 10,000 cycles under 0.5% strain.

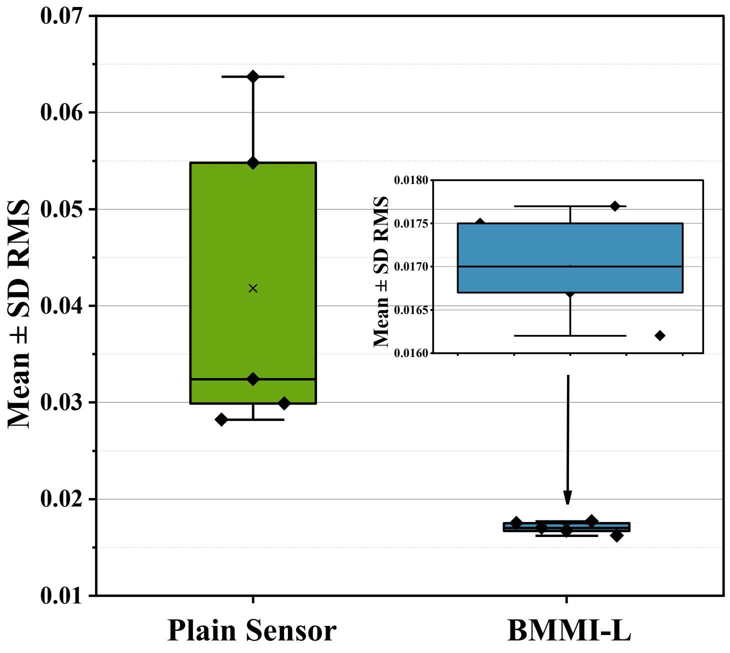

Fig. S15. Quantitative comparison of artefacts-induced signal fluctuations between the plain sensor and BMMI-L. Root-mean-square (RMS) amplitude of the relative resistance signal ($\Delta R/R_0$) measured for the plain sensor and the BMMI-L sensor (mean±SD, n=5). The BMMI-L sensor (0.0170 ± 0.00054) exhibits a lower RMS level with reduced variability compared with the plain sensor (0.0418 ± 0.0146), indicating excellent resistance to motion artefacts.

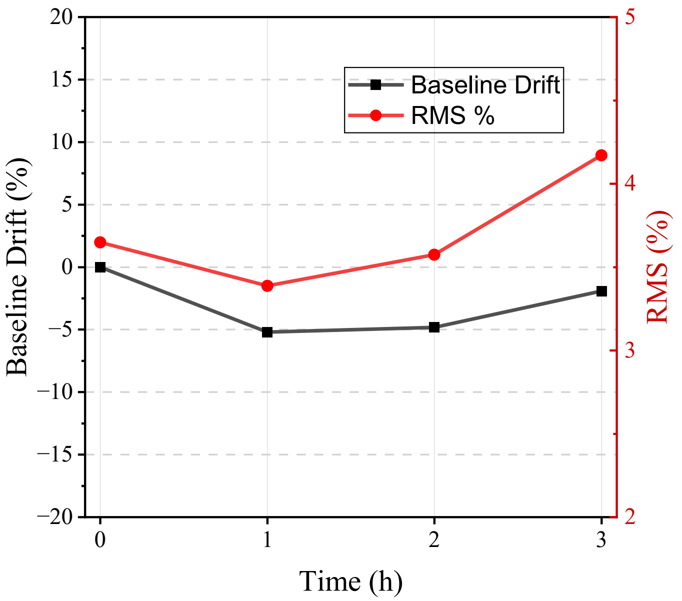

Fig. S16. Baseline drift and RMS noise of the BMMI during prolonged on-skin wear. The baseline resistance drift (left axis) and RMS noise level (right axis) were evaluated over 3 h of continuous skin contact.

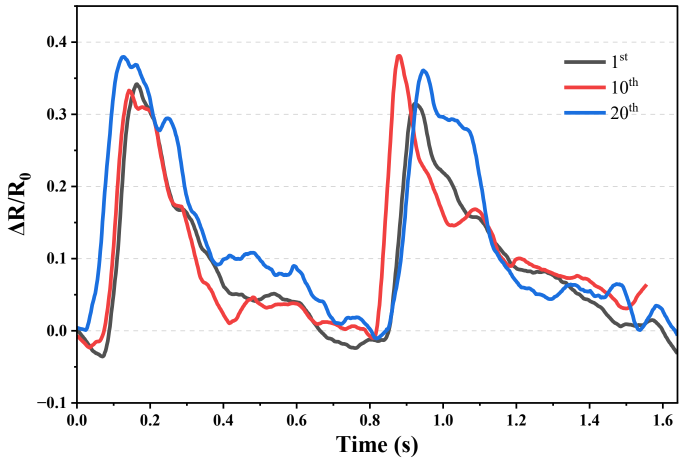

Fig. S17. Representative pulse waveforms measured after the 1st, 10th, and 20th attachment of the BMMI to the same skin location. The consistent waveform shapes and preserved fine features demonstrate stable skin–device adhesion and reliable signal fidelity under repeated attachment/detachment cycles.

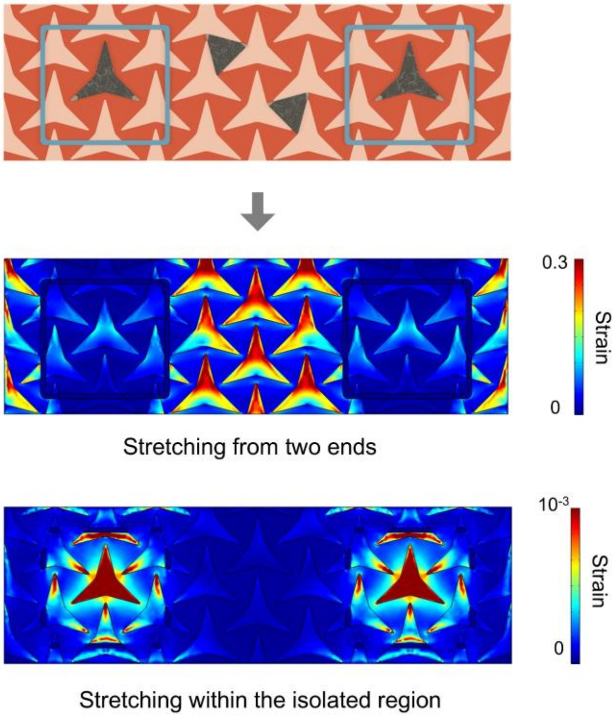

Fig. S18. FEA results of the BMMI with isolation frames under stretching applied either from the two ends or within the isolated regions. When tensile strain is applied from both ends, the strain within the isolated region enclosed by the frames is significantly reduced. At the same time, high sensitivity is maintained inside the frame.

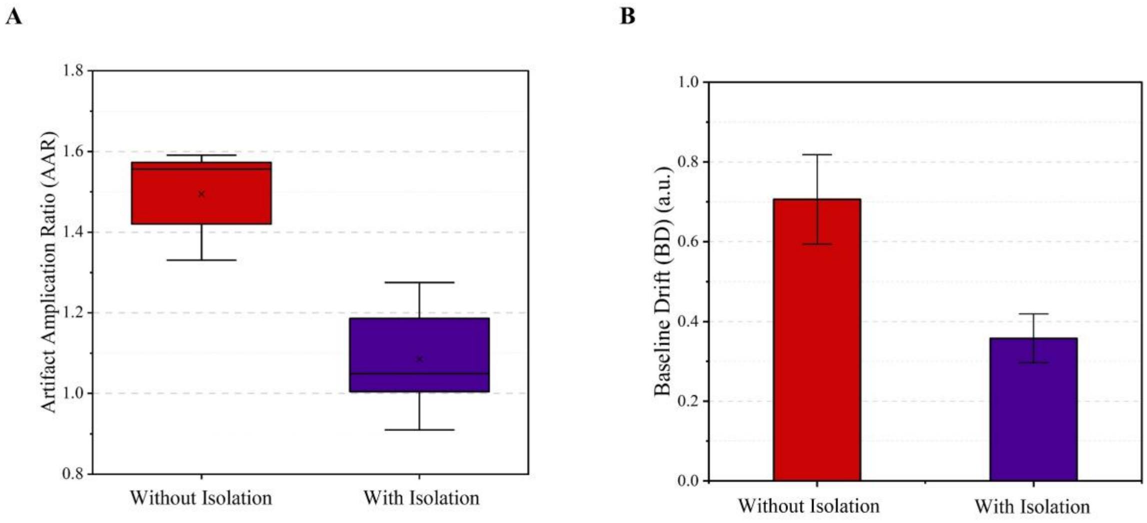

Fig. S19. Quantitative analysis of isolation effectiveness for BMMI-S. (A) Comparison of artifact amplification ratio (AAR) with and without isolation during head turning. (B) Comparison of baseline drift (BD) with and without isolation during head turning. The results demonstrate that the isolation structure reduces the AAR from 1.494 ± 0.102 to 1.085 ± 0.130, corresponding to an approximate 82.79% reduction, and decreases the BD from 0.706 ± 0.112 to 0.358 ± 0.061, corresponding to an approximate 49.29% reduction. (mean±SD, n=5)

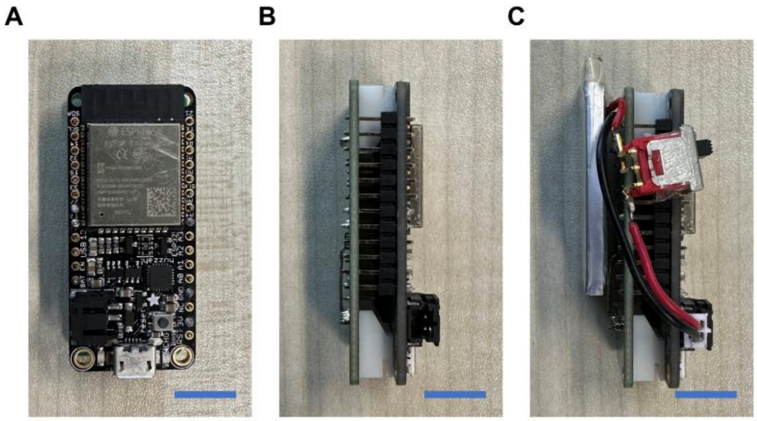

Fig. S20. Wireless readout module of BMMI. (A) ESP32 microcontroller featuring high-speed data processing and Bluetooth Low Energy (BLE) wireless transmission capabilities. (B) ESP32 integrated with a 1-to-4 analog multiplexer for multi-channel signal acquisition. (C) The readout module is powered by a 3.7 V, 350 mAh lithium polymer (LiPo) battery. Scale bar 1cm.

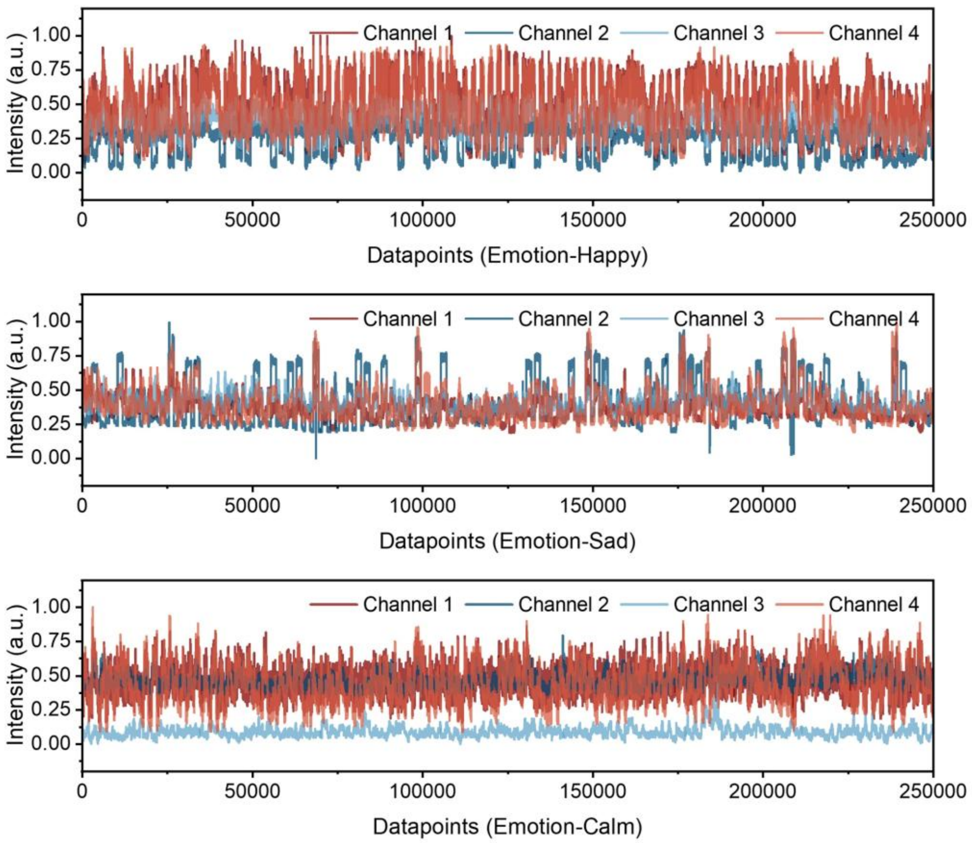

Fig. S21. Datasets for emotion classification. 3 emotion datasets collected by wireless 4-channel BMMI, comprising 250,000 data points per emotion, segmented into 100 groups of 2,500 data points each.

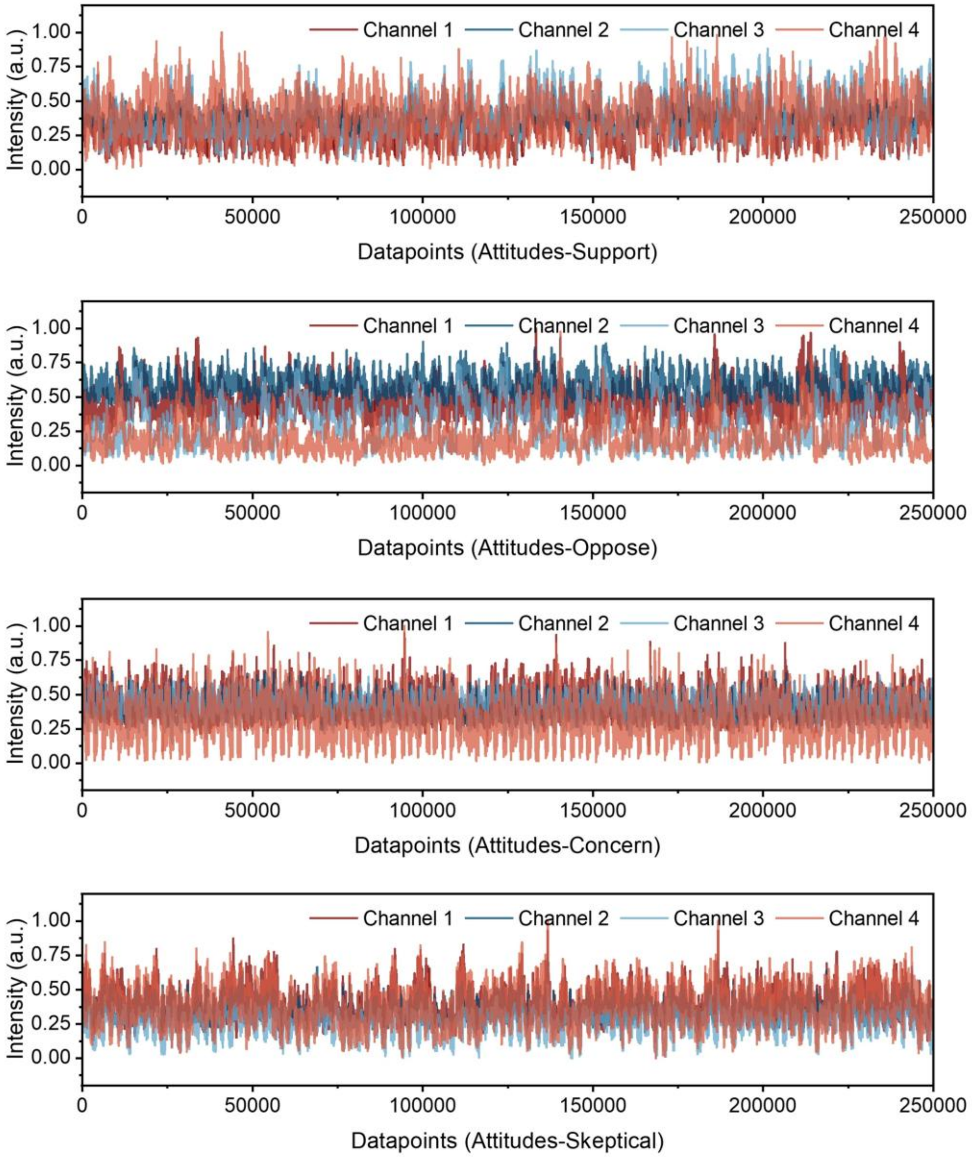

Fig. S22. Datasets for attitude classification. 4 attitudes datasets collected by wireless 4-channel BMMI, comprising 250,000 data points per attitude, segmented into 100 groups of 2,500 data points each.

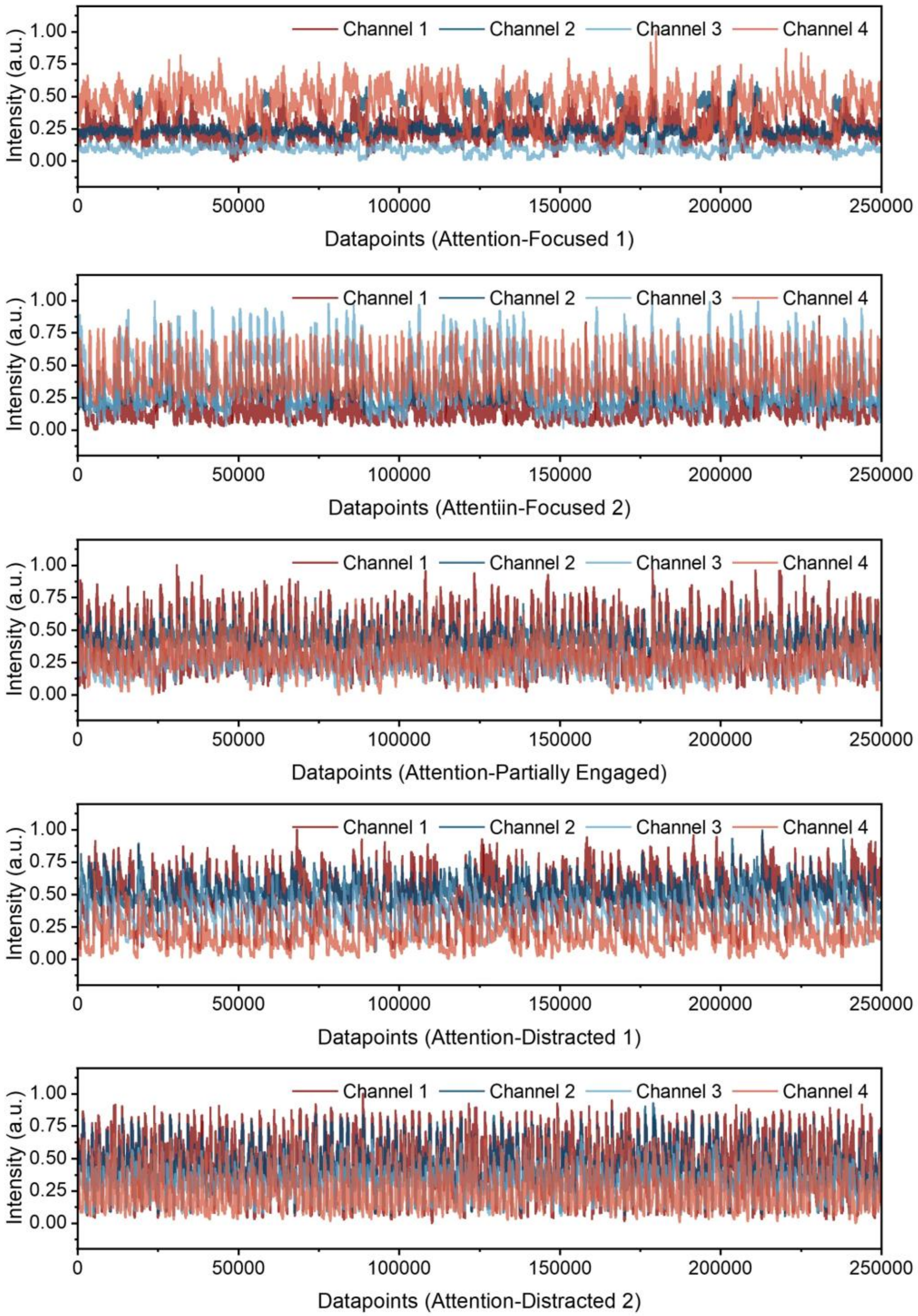

Fig. S23. Datasets for attention classification. 5 attitudes datasets collected by wireless 4-channel BMMI, comprising 250,000 data points per attitude, segmented into 100 groups of 2,500 data points each.

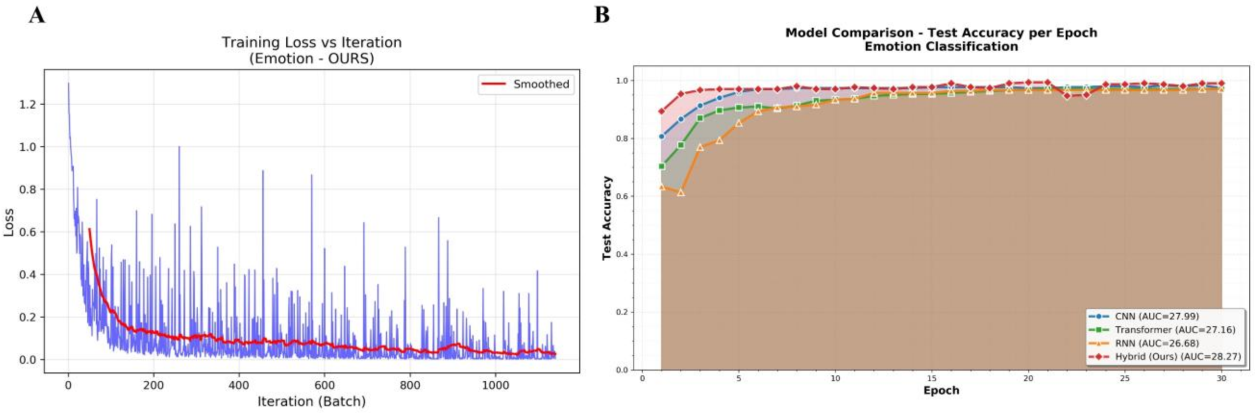

Fig. S24. (A) Training loss curves of our CA-Net model in emotion recognition. (B) Comparison of test accuracy curves across epochs between our hybrid CA-Net and other models in emotion recognition.

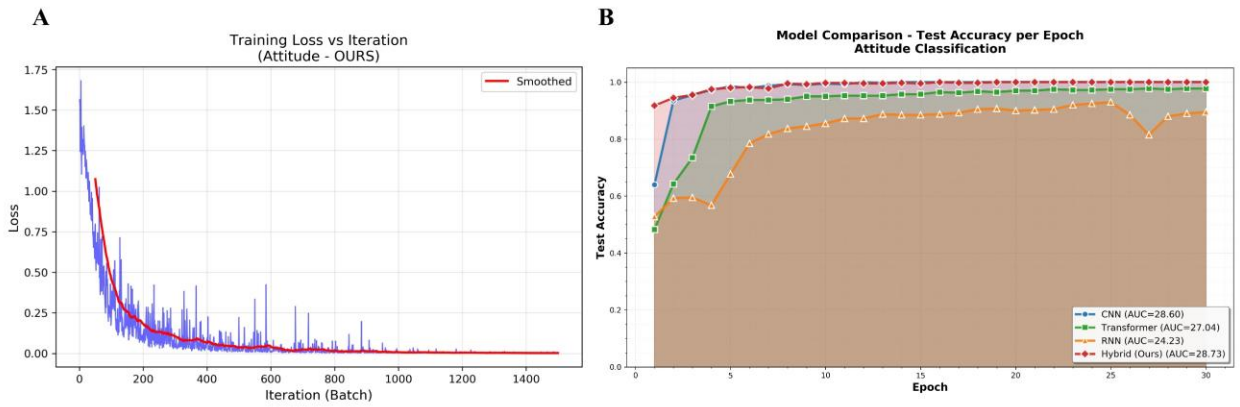

Fig. S25. (A) Training loss curves of our CA-Net model in attitudes recognition. (B) Comparison of test accuracy curves across epochs between our hybrid CA-Net and other models in attitudes recognition.

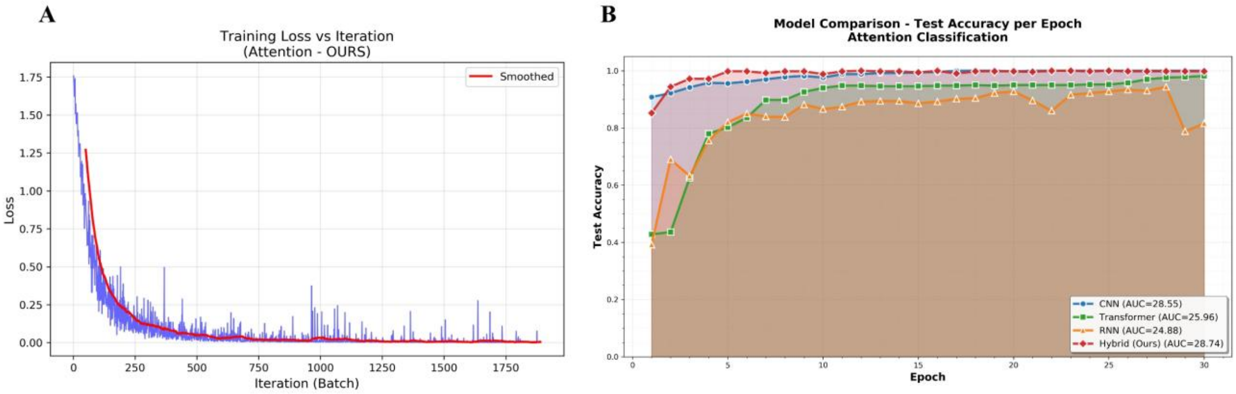

Fig. S26. (A) Training loss curves of our CA-Net model in attention recognition. (B) Comparison of test accuracy curves across epochs between our hybrid CA-Net and other models in attention recognition.

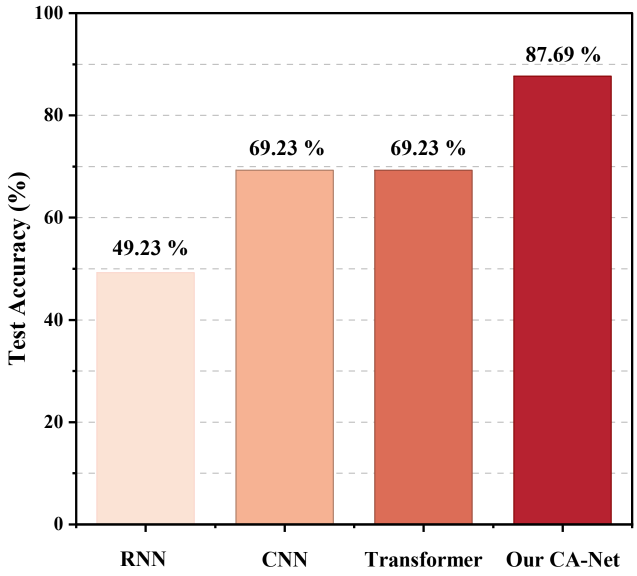

Fig. S27. Comparison of recognition accuracy on 12 cognitive and affective states for new users. A total of 65 experimental sessions comprising 650,000 data points were collected from three new subjects across different age groups (30s, 40s, and 50s) to evaluate the generalization capability of the proposed technology.

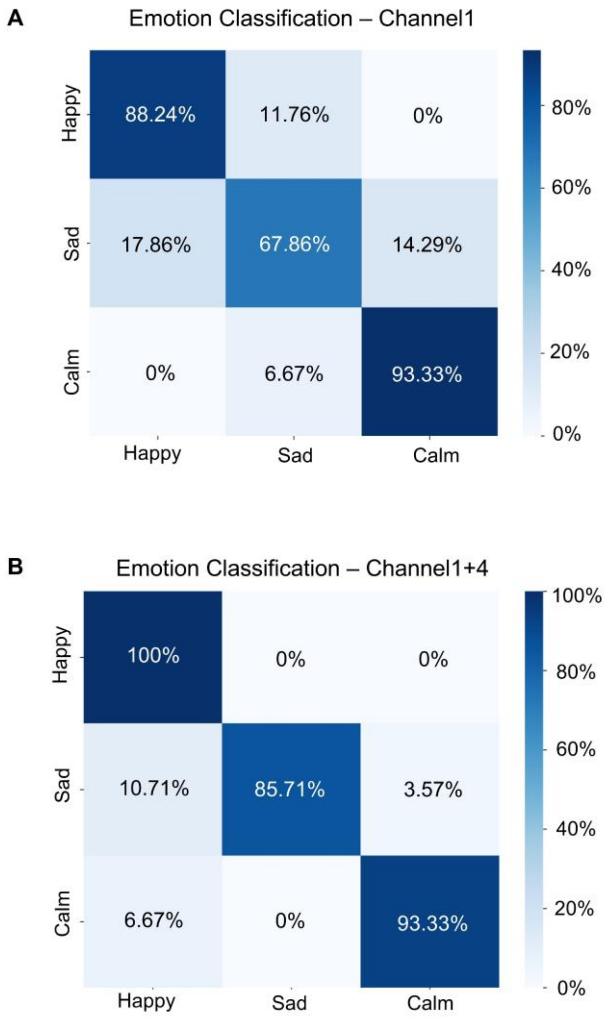

Fig. S28. Confusion matrices for emotion classification in the ablation study. (A) Emotion classification using the Channel 1 signal, achieving 80% accuracy. (B) Emotion classification using signals from Channels 1 and 4, achieving 91.67% accuracy.

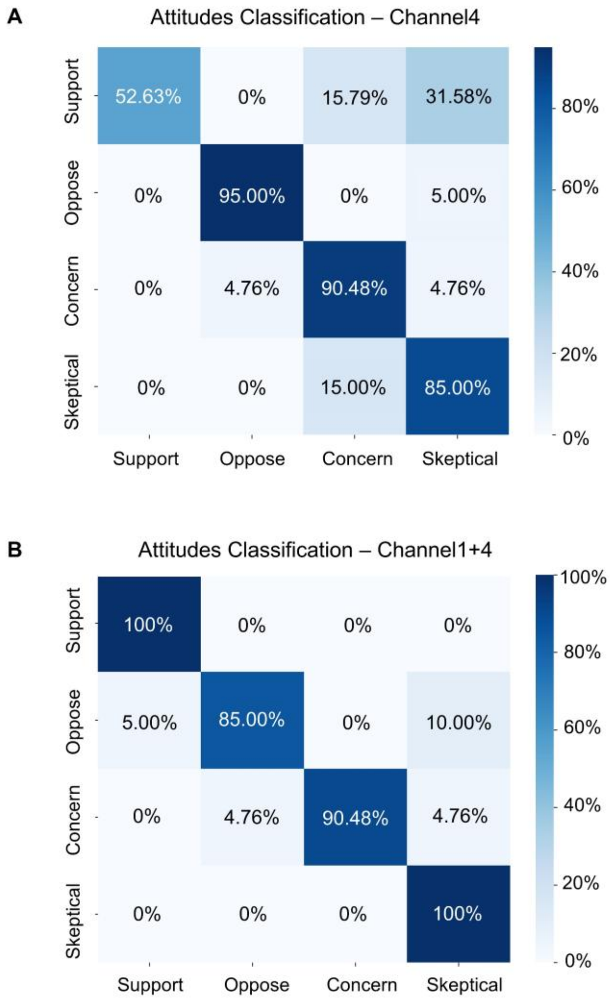

Fig. S29. Confusion matrices for attitude classification in the ablation study. (A) Attitude classification using the Channel 4 signal, achieving 81.25% accuracy. (B) Attitude classification using the signals from Channels 1 and 4, achieving 93.75% accuracy.

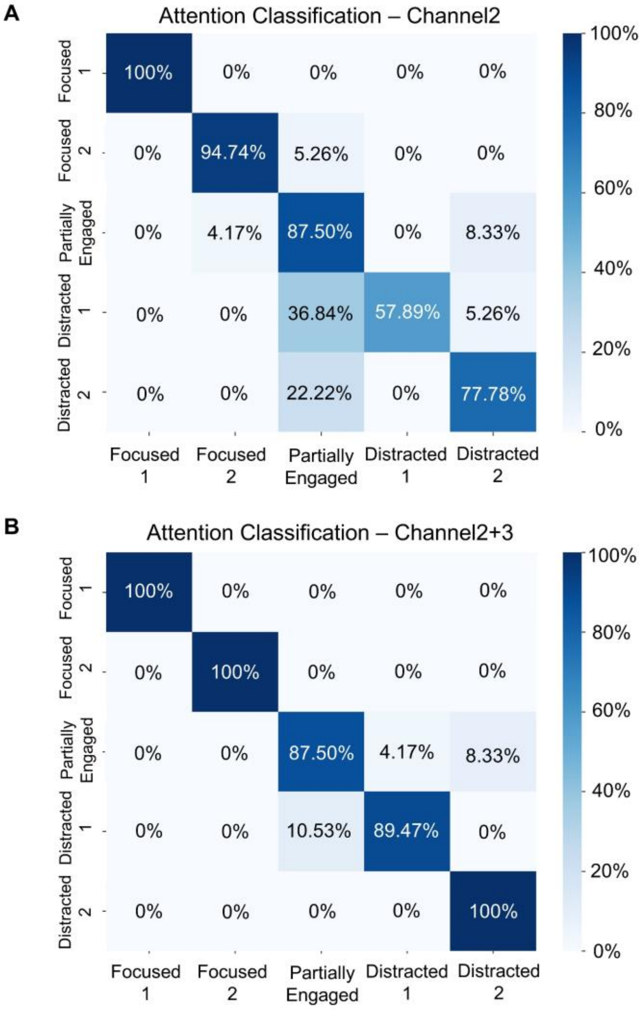

Fig. S30. Confusion matrices for attention classification in the ablation study. (A) Attention classification using the Channel 2 signal, achieving 84% accuracy. (B) Attention classification using the signals from Channels 2 and 3, achieving 95% accuracy.

Ref	Sensing Mechanism	Interface Structure	Sensitivity Tunability	Dual Sensitivity	Sensitivity (Gauge Factor)	Detection Limit	Response Time	Durability	Classification	Accuracy
**(*57*)**	Piezoresistive	Fibrous mat	×	×	10	0.05 %	60 ms	2000 cycles	/	/
**(*58*)**	Piezoresistive	Fibrous mat	×	×	86.9	/	148.9 ms	8500 cycles	5 Channels Facial expression	98.3 %
**(*59*)**	Piezoresistive	Flat	✓	×	1443~9039	/	/	1000 cycles	/	/
**(*60*)**	Piezoresistive	Flat	×	×	1187	/	60 ms	2000 cycles	/	/
**(*61*)**	Piezoresistive	Serpentine meander structure	×	×	1000	0.04 %	58 ms	10000 cycles	5 Channels Sign language	98 %
**(*26*)**	Piezoresistive	Serpentine structures	×	×	100	/	/	50000 cycles	4 Channels Words	87.53 %
**(*62*)**	Piezoresistive	Auxetic Metamaterial	×	×	177 (Region I)	/	/	3000 cycles	/	/
**(*63*)**	Piezoresistive	Auxetic Metamaterial	×	×	835	/	/	2000 cycles	/	/
**BMMI**	Piezoresistive	Biomimetic Auxetic Metamaterial	✓	✓	26~1186	0.01 ~ 0.1 %	15 ms	10000 cycles	4 Channels Cognitive & Affects	98.33 %

Table S1. Comparison of the BMMI with representative skin-mounted piezoresistive interfaces (*26, 57-63*).

Dataset	Backbone	Accuracy	Precision	Recall
Emotion	CA-Net	0.9500	0.9544	0.9567
	RNN	0.9167	0.9123	0.9275
	Transformer	0.9167	0.9306	0.9123
Attitudes	CA-Net	1.0000	1.0000	1.0000
	RNN	0.9000	0.8851	0.8897
	Transformer	1.0000	1.0000	1.0000
Attention	CA-Net	0.9900	0.9920	0.9895
	RNN	0.9200	0.9139	0.9152
	Transformer	0.9700	0.9739	0.9727

Table S2. Classification results of comparative experiments between our CA-Net, conventional RNN, and pure Transformer-based models using four-channel signals collected by the wireless BMMI.

Ref	Device	Sensing Mechanism	Sensor Location	Sensing Target	Number of Channels	Classification method	Number of states	Accuracy
**(*64*)**	Commercial bracelet (Algoband F8)	PPG	Wrist	Heart rate	1	RF, KNN,DT, GBDT	3	81.2%
**(*65*)**	EEG electrodes	EEG	Brain	Brain electrical activity	40	QDC+RNN	9	89%
**(*66*)**	EMG electrodes	EMG	Face	Facial muscle activity	8	KNN,SVM,LDA	6	96.3%
**(*67*)**	Skin-integrated facial interface	Triboelectric sensing	Face + Neck	Small vibration + Large deformation	6	CNN	5	93.3 %
**(*68*)**	Textile strain sensors	Piezoresistive sensing	Neck	Small vibration	2	1D-CNN with DFT	3	83.2%
**BMMI System**	Biomimetic Metamaterial-based Interface	Dual Tuneable Piezoresistive Sensing	Neck	Small vibration + Large deformation	4	CA-Net	12	98.33 %

Table S3. Comparison of the BMMI with wearable-sensor-based approaches for affective and cognitive state recognition (*64-68*).

Movie S1.
BMMI Functional Substrate under Different Strains.

Movie S2.
BMMI-S and BMMI-L Sensors under Tensile Strain.

Movie S3.
Wireless BMMI System for Cognitive and Affective States Recognition.

REFERENCES

1. S. Lee, S. Franklin, F. A. Hassani, T. Yokota, M. O. G. Nayeem, Y. Wang, R. Leib, G. Cheng, D. W. Franklin, T. Someya, Nanomesh pressure sensor for monitoring finger manipulation without sensory interference. *Science* **370**, 966–970 (2020).

2. Q. Yang, W. Jin, Q. Zhang, Y. Wei, Z. Guo, X. Li, Y. Yang, Q. Luo, H. Tian, T.-L. Ren, Mixed-modality speech recognition and interaction using a wearable artificial throat. *Nat. Mach. Intell.* **5**, 169–180 (2023).

3. O. A. Araromi, M. A. Graule, K. L. Dorsey, S. Castellanos, J. R. Foster, W.-H. Hsu, A. E. Passy, J. J. Vlassak, J. C. Weaver, C. J. Walsh, R. J. Wood, Ultra-sensitive and resilient compliant strain gauges for soft machines. *Nature* **587**, 219–224 (2020).

4. Q. Wang, H. Guan, C. Wang, P. Lei, H. Sheng, H. Bi, J. Hu, C. Guo, Y. Mao, J. Yuan, M. Shao, Z. Jin, J. Li, W. Lan, A wireless, self-powered smart insole for gait monitoring and recognition via nonlinear synergistic pressure sensing. *Sci. Adv.* **11**, eadu1598 (2025).

5. Y. Kim, J. M. Suh, J. Shin, Y. Liu, H. Yeon, K. Qiao, H. S. Kum, C. Kim, H. E. Lee, C. Choi, H. Kim, D. Lee, J. Lee, J.-H. Kang, B.-I. Park, S. Kang, J. Kim, S. Kim, J. A. Perozek, K. Wang, Y. Park, K. Kishen, L. Kong, T. Palacios, J. Park, M.-C. Park, H. Kim, Y. S. Lee, K. Lee, S.-H. Bae, W. Kong, J. Han, J. Kim, Chip-less wireless electronic skins by remote epitaxial freestanding compound semiconductors. *Science* **377**, 859–864 (2022).

6. K. K. Kim, M. Kim, K. Pyun, J. Kim, J. Min, S. Koh, S. E. Root, J. Kim, B.-N. T. Nguyen, Y. Nishio, S. Han, J. Choi, C.-Y. Kim, J. B.-H. Tok, S. Jo, S. H. Ko, Z. Bao, A substrate-less nanomesh receptor with meta-learning for rapid hand task recognition. *Nat. Electron.* **6**, 64–75 (2023).

7. C. Tang, W. Yi, M. Xu, Y. Jin, Z. Zhang, X. Chen, C. Liao, M. Kang, S. Gao, P. Smielewski, L. G. Occhipinti, A deep learning–enabled smart garment for accurate and versatile monitoring of sleep conditions in daily life. *Proc. Natl. Acad. Sci. U.S.A.* **122**, e2420498122 (2025).

8. Z. Zhou, K. Chen, X. Li, S. Zhang, Y. Wu, Y. Zhou, K. Meng, C. Sun, Q. He, W. Fan, E. Fan, Z. Lin, X. Tan, W. Deng, J. Yang, J. Chen, Sign-to-speech translation using machine-learning-assisted stretchable sensor arrays. *Nat. Electron.* **3**, 571–578 (2020).

9. M. Xu, J. Zhang, C. Dong, C. Tang, F. Hu, G. G. Malliaras, L. G. Occhipinti, Simultaneous isotropic omnidirectional hypersensitive strain sensing and deep learning-assisted direction recognition in a biomimetic stretchable device. *Adv. Mater.* **37**, 2420322 (2025).

10. S. Li, H. Wang, W. Ma, L. Qiu, K. Xia, Y. Zhang, H. Lu, M. Zhu, X. Liang, X.-E. Wu, H. Liang, Y. Zhang, Monitoring blood pressure and cardiac function without positioning via a deep learning–assisted strain sensor array. *Sci. Adv.* **9**, eadh0615 (2023).

11. T. D. Royce, W. L. Bowcher, Eds., *New Directions in the Analysis of Multimodal Discourse* (L. Erlbaum Associates, 2007).

12. S. Gong, X. Zhang, X. A. Nguyen, Q. Shi, F. Lin, S. Chauhan, Z. Ge, W. Cheng, Hierarchically resistive skins as specific and multimetric on-throat wearable biosensors. *Nat. Nanotechnol.* **18**, 889–897 (2023).

13. D. H. Lee, T. Miyashita, Y. Xuan, K. Takei, Ultrasensitive and stretchable strain sensors based on laser-induced graphene with ZnO nanoparticles. *ACS Nano* **18**, 32255–32265 (2024).

14. S. Zhao, X. Meng, L. Liu, W. Bo, M. Xia, R. Zhang, D. Cao, J.-H. Ahn, Polypyrrole-coated copper nanowire-threaded silver nanoflowers for wearable strain sensors with high sensing performance. *Chem. Eng. J.* **417**, 127966 (2021).

15. D. Kang, P. V. Pikhitsa, Y. W. Choi, C. Lee, S. S. Shin, L. Piao, B. Park, K.-Y. Suh, T. Kim, M. Choi, Ultrasensitive mechanical crack-based sensor inspired by the spider sensory system. *Nature* **516**, 222–226 (2014).

16. C. Tang, M. Xu, W. Yi, Z. Zhang, E. Occhipinti, C. Dong, D. Ravenscroft, S.-M. Jung, S. Lee, S. Gao, J. M. Kim, L. G. Occhipinti, Ultrasensitive textile strain sensors redefine wearable silent speech interfaces with high machine learning efficiency. *NPJ Flexible Electron.* **8**, 27 (2024).

17. X. Huang, L. Liu, Y. H. Lin, R. Feng, Y. Shen, Y. Chang, H. Zhao, High-stretchability and low-hysteresis strain sensors using origami-inspired 3D mesostructures. *Sci. Adv.* **9**, eadh9799 (2023).

18. L. Li, Y. Zheng, E. Liu, X. Zhao, S. Yu, J. Wang, X. Han, F. Xu, Y. Cao, C. Lu, H. Gao, Stretchable and ultrasensitive strain sensor based on a bilayer wrinkle-microcracking mechanism. *Chem. Eng. J.* **437**, 135399 (2022).

19. A. Handler, D. D. Ginty, The mechanosensory neurons of touch and their mechanisms of activation. *Nat. Rev. Neurosci.* **22**, 521–537 (2021).

20. T. Li, H. Qi, C. Zhao, Z. Li, W. Zhou, G. Li, H. Zhuo, W. Zhai, Robust skin-integrated conductive biogel for high-fidelity detection under mechanical stress. *Nat. Commun.* **16**, 88 (2025).

21. G. Limbert, E. Kuhl, On skin microrelief and the emergence of expression micro-wrinkles. *Soft Matter* **14**, 1292–1300 (2018).

22. W. Sun, B. Wang, T. Yang, R. Yin, F. Wang, H. Zhang, W. Zhang, Three-dimensional bioprinted skin microrelief and its role in skin aging. *Biomimetics* **9**, 366 (2024).

23. A. Zimmerman, L. Bai, D. D. Ginty, The gentle touch receptors of mammalian skin. *Science* **346**, 950–954 (2014).

24. R. S. Johansson, J. R. Flanagan, Coding and use of tactile signals from the fingertips in object manipulation tasks. *Nat. Rev. Neurosci.* **10**, 345–359 (2009).

25. A. Chortos, J. Liu, Z. Bao, Pursuing prosthetic electronic skin. *Nat. Mater.* **15**, 937–950 (2016).

26. T. Kim, Y. Shin, K. Kang, K. Kim, G. Kim, Y. Byeon, H. Kim, Y. Gao, J. R. Lee, G. Son, T. Kim, Y. Jun, J. Kim, J. Lee, S. Um, Y. Kwon, B. G. Son, M. Cho, M. Sang, J. Shin, K. Kim, J. Suh, H. Choi, S. Hong, H. Cheng, H.-G. Kang, D. Hwang, K. J. Yu, Ultrathin crystalline-silicon-based strain gauges with deep learning algorithms for silent speech interfaces. *Nat. Commun.* **13**, 5815 (2022).

27. T. Liu, M. Zhang, Z. Li, H. Dou, W. Zhang, J. Yang, P. Wu, D. Li, X. Mu, Machine learning-assisted wearable sensing systems for speech recognition and interaction. *Nat. Commun.* **16**, 2363 (2025).

28. T. Frenzel, M. Kadic, M. Wegener, Three-dimensional mechanical metamaterials with a twist. *Science* **358**, 1072–1074 (2017).

29. J. B. Berger, H. N. G. Wadley, R. M. McMeeking, Mechanical metamaterials at the theoretical limit of isotropic elastic stiffness. *Nature* **543**, 533–537 (2017).

30. X. Fang, J. Wen, L. Cheng, D. Yu, H. Zhang, P. Gumbsch, Programmable gear-based mechanical metamaterials. *Nat. Mater.* **21**, 869–876 (2022).

31. Q. Zeng, X. Tian, D. T. Nguyen, C. Li, P. Chia, B. C. K. Tee, C. Wu, J. S. Ho, A digitally embroidered metamaterial biosensor for kinetic environments. *Nat. Electron.* **7**, 1025–1034 (2024).

32. D. Hwang, C. Lee, X. Yang, J. M. Pérez-González, J. Finnegan, B. Lee, E. J. Markvicka, R. Long, M. D. Bartlett, Metamaterial adhesives for programmable adhesion through reverse crack propagation. *Nat. Mater.* **22**, 1030–1038 (2023).

33. S. Jiang, X. Liu, J. Liu, D. Ye, Y. Duan, K. Li, Z. Yin, Y. Huang, Flexible metamaterial electronics. *Adv. Mater.* **34**, 2200070 (2022).

34. K. Bertoldi, V. Vitelli, J. Christensen, M. van Hecke, Flexible mechanical metamaterials. *Nat. Rev. Mater.* **2**, 17066 (2017).

35. H. M. A. Kolken, A. A. Zadpoor, Auxetic mechanical metamaterials. *RSC Adv.* **7**, 5111–5129 (2017).

36. Y. Jiang, Z. Liu, C. Wang, X. Chen, Heterogeneous strain distribution of elastomer substrates to enhance the sensitivity of stretchable strain sensors. *Acc. Chem. Res.* **52**, 82–90 (2019).

37. J. N. Grima, R. Gatt, B. Ellul, E. Chetcuti, Auxetic behaviour in non-crystalline materials having star or triangular shaped perforations. *J. Non Cryst. Solids* **356**, 1980–1987 (2010).

38. S. Min, J. An, J. H. Lee, J. H. Kim, D. J. Joe, S. H. Eom, C. D. Yoo, H.-S. Ahn, J.-Y. Hwang, S. Xu, J. A. Rogers, K. J. Lee, Wearable blood pressure sensors for cardiovascular monitoring and machine learning algorithms for blood pressure estimation. *Nat. Rev. Cardiol.* **22**, 629–648 (2025).

39. M. T. V. Yamuza, J. Bolea, M. Orini, P. Laguna, C. Orrite, M. Vallverdú, R. Bailón, Human emotion characterization by heart rate variability analysis guided by respiration. *IEEE J. Biomed. Health Inform.* **23**, 2446–2454 (2019).

40. B. M. Appelhans, L. J. Luecken, Heart rate variability as an index of regulated emotional responding. *Rev. Gen. Psychol.* **10**, 229–240 (2006).

41. K. Yang, C. Wang, Y. Gu, Z. Sarsenbayeva, B. Tag, T. Dingler, G. Wadley, J. Goncalves, Behavioral and physiological signals-based deep multimodal approach for mobile emotion recognition. *IEEE Trans. Affect. Comput.* **14**, 1082–1097 (2023).

42. Y. Hu, B. Chen, J. Lin, Y. Wang, Y. Wang, C. Mehlman, H. Lipson, Human-robot facial coexpression. *Sci. Robot.* **9**, eadi4724 (2024).

43. K. He, X. Zhang, S. Ren, J. Sun, "Deep residual learning for image recognition," in *Proceedings of the IEEE Conference on Computer Vision and Pattern Recognition* (IEEE, 2016), pp. 770–778. http://openaccess.thecvf.com/content_cvpr_2016/html/He_Deep_Residual_Learning_CVPR_2016_paper.html.

44. A. Vaswani, N. Shazeer, N. Parmar, J. Uszkoreit, L. Jones, A. N. Gomez, Ł. ukasz Kaiser, I. Polosukhin, "Attention is all you need," in *Advances in Neural Information Processing Systems* (Curran Associates Inc., 2017), vol. 30. https://proceedings.neurips.cc/paper/2017/hash/3f5ee243547dee91fbd053c1c4a845aa-Abstract.html.

45. J. Zhang, Y. Hu, X. Qi, T. Meng, L. Wang, H. Fu, M. Yang, J. Liu, "Polar eyeball shape net for 3D posterior ocular shape representation," in *Medical Image Computing and Computer Assisted Intervention – MICCAI 2023*, H. Greenspan, A. Madabhushi, P. Mousavi, S. Salcudean, J. Duncan, T. Syeda-Mahmood, R. Taylor, Eds. (Springer Nature Switzerland,

2023), vol. 14225 of *Lecture Notes in Computer Science*, pp. 180–190. https://link.springer.com/10.1007/978-3-031-43987-2_18.

46. J. Zhang, X. Wu, J. Liu, C. Zou, F. Nie, Z. Sun, X. Qi, J. Liu, Polar subarea-aware fusion net for posterior eyeball shape reconstruction. *IEEE Trans. Med. Imaging* , 1 (2025).

47. A. Sharfeddin, A. A. Volinsky, G. Mohan, N. D. Gallant, Comparison of the macroscale and microscale tests for measuring elastic properties of polydimethylsiloxane. *J. Appl. Polym. Sci.* **132**, app.42680 (2015).

48. I. D. Johnston, D. K. McCluskey, C. K. L. Tan, M. C. Tracey, Mechanical characterization of bulk Sylgard 184 for microfluidics and microengineering. *J. Micromech. Microeng.* **24**, 035017 (2014).

49. Y.-S. Yu, Y.-P. Zhao, Deformation of PDMS membrane and microcantilever by a water droplet: Comparison between Mooney–Rivlin and linear elastic constitutive models. *J. Colloid Interface Sci.* **332**, 467–476 (2009).

50. O. Akogwu, D. Kwabi, S. Midturi, M. Eleruja, B. Babatope, W. O. Soboyejo, Large strain deformation and cracking of nano-scale gold films on PDMS substrate. *Mater. Sci. Eng. B* **170**, 32–40 (2010).

51. A. E. Forte, P. Z. Hanakata, L. Jin, E. Zari, A. Zareei, M. C. Fernandes, L. Sumner, J. Alvarez, K. Bertoldi, Inverse design of inflatable soft membranes through machine learning. *Adv. Funct. Mater.* **32**, 2111610 (2022).

52. Y. Yu, D. Sanchez, N. Lu, Work of adhesion/separation between soft elastomers of different mixing ratios. *J. Mater. Res.* **30**, 2702–2712 (2015).

53. M. P. Wolf, G. B. Salieb-Beugelaar, P. Hunziker, PDMS with designer functionalities—Properties, modifications strategies, and applications. *Prog. Polym. Sci.* **83**, 97–134 (2018).

54. B. Ruben, M. Elisa, L. Leandro, M. Victor, G. Gloria, S. Marina, S. M. K, R. Pandiyan, L. Nadhira, Oxygen plasma treatments of polydimethylsiloxane surfaces: Effect of the atomic oxygen on capillary flow in the microchannels. *Micro Nano Lett.* **12**, 754–757 (2017).

55. A. Borók, K. Laboda, A. Bonyár, PDMS bonding technologies for microfluidic applications: A review. *Biosensors* **11**, 292 (2021).

56. L. Francioso, C. De Pascali, R. Bartali, E. Morganti, L. Lorenzelli, P. Siciliano, N. Laidani, PDMS/Kapton interface plasma treatment effects on the polymeric package for a wearable thermoelectric generator. *ACS Appl. Mater. Interfaces* **5**, 6586–6590 (2013).

57. H. Li, J. Chen, X. Chang, Y. Xu, G. Zhao, Y. Zhu, Y. Li, A highly stretchable strain sensor with both an ultralow detection limit and an ultrawide sensing range. *J. Mater. Chem. A* **9**, 1795–1802 (2021).

58. X. Zong, C. Zhang, N. Zhang, Z. Wang, J. Wang, Breathable, superhydrophobic and multifunctional Janus nanofibers for dual-mode passive thermal management/facial expression recognition with deep learning. *Chem. Eng. J.* **505**, 159759 (2025).

59. C. Yang, W. Huang, Y. Lin, S. Cao, H. Wang, Y. Sun, T. Fang, M. Wang, D. Kong, Stretchable MXene/carbon nanotube bilayer strain sensors with tunable sensitivity and working ranges. *ACS Appl. Mater. Interfaces* **16**, 30274–30283 (2024).

60. H. Zhang, D. Zhang, J. Guan, D. Wang, M. Tang, Y. Ma, H. Xia, A flexible wearable strain sensor for human-motion detection and a human–machine interface. *J. Mater. Chem. C* **10**, 15554–15564 (2022).

61. X. Wu, X. Luo, Z. Song, Y. Bai, B. Zhang, G. Zhang, Ultra-robust and sensitive flexible strain sensor for real-time and wearable sign language translation. *Adv. Funct. Mater.* **33**, 2303504 (2023).

62. T. Hu, T. Pan, D. Guo, Y. Xiao, F. Li, M. Gao, Z. Huang, J. Zhu, T. Cheng, Y. Lin, Omnidirectional configuration of stretchable strain sensor enabled by the strain engineering with chiral auxetic metamaterial. *ACS Nano* **17**, 22035–22045 (2023).

63. Y. Jiang, Z. Liu, N. Matsuhisa, D. Qi, W. R. Leow, H. Yang, J. Yu, G. Chen, Y. Liu, C. Wan, Z. Liu, X. Chen, Auxetic mechanical metamaterials to enhance sensitivity of stretchable strain sensors. *Adv. Mater.* **30**, 1706589 (2018).

64. L. Shu, Y. Yu, W. Chen, H. Hua, Q. Li, J. Jin, X. Xu, Wearable emotion recognition using heart rate data from a smart bracelet. *Sensors* **20**, 718 (2020).

65. S. Gannouni, A. Aledaily, K. Belwafi, H. Aboalsamh, Emotion detection using electroencephalography signals and a zero-time windowing-based epoch estimation and relevant electrode identification. *Sci. Rep.* **11**, 7071 (2021).

66. M. Kołodziej, A. Majkowski, M. Jurczak, Acquisition and analysis of facial electromyographic signals for emotion recognition. *Sensors* **24**, 4785 (2024).

67. J. P. Lee, H. Jang, Y. Jang, H. Song, S. Lee, P. S. Lee, J. Kim, Encoding of multi-modal emotional information via personalized skin-integrated wireless facial interface. *Nat. Commun.* **15**, 530 (2024).

68. C. Tang, S. Gao, C. Li, W. Yi, Y. Jin, X. Zhai, S. Lei, H. Meng, Z. Zhang, M. Xu, S. Wang, X. Chen, C. Wang, H. Yang, N. Wang, W. Wang, J. Cao, X. Feng, P. Smielewski, Y. Pan, W. Song, M. Birchall, L. G. Occhipinti, Wearable intelligent throat enables natural speech in stroke patients with dysarthria. *Nat. Commun.* **17**, 293 (2026).
